# Transformable prodrug nanoplatform *via* tumor microenvironment modulation and immune checkpoint blockade potentiates immunogenic cell death mediated cancer immunotherapy

**DOI:** 10.7150/thno.83912

**Published:** 2023-03-21

**Authors:** Weijing Yang, Jinmeng Yi, Rongrong Zhu, Yichen Guo, Kaixin Zhang, Yongjian Cao, Xinyan Li, Jinjie Zhang, Zhenzhong Zhang, Yongjuan Li, Xiaoyuan Chen

**Affiliations:** 1 School of Pharmaceutical Sciences, Zhengzhou University, Zhengzhou 450001, China; 2 Key Laboratory of Targeting Therapy and Diagnosis for Critical Diseases, Zhengzhou 450001, Henan Province, China; 3 The center of Infection and Immunity, Academy of Medical Sciences, Zhengzhou University, Zhengzhou, Henan 450001, China; 4 Medical Research Center, The First Affiliated Hospital of Zhengzhou University, Zhengzhou University, Zhengzhou, Henan 450001, China; 5 Departments of Diagnostic Radiology, Chemical and Biomolecular Engineering, and Biomedical Engineering, Yong Loo Lin School of Medicine and Faculty of Engineering, National University of Singapore, Singapore, 117597 Singapore; 6 Clinical Imaging Research Centre, Centre for Translational Medicine, Yong Loo Lin School of Medicine, National University of Singapore, Singapore 117599, Singapore; 7 Nanomedicine Translational Research Program, NUS Center for Nanomedicine, Yong Loo Lin School of Medicine, National University of Singapore, Singapore 117597, Singapore

**Keywords:** polymersome-micelle transformable nanoplatform, tumor microenvironment modulation, immune checkpoint blockade, immunogenic cell death, cancer immunotherapy

## Abstract

**Rationale:** Chemoimmunotherapy is a promising approach in cancer immunotherapy. However, its therapeutic efficacy is restricted by high reactive oxygen species (ROS) levels, an abundance of cancer-associated fibroblasts (CAFs) in tumor microenvironment (TME) as well as immune checkpoints for escaping immunosurveillance.

**Methods:** Herein, a new type of TME and reduction dual-responsive polymersomal prodrug (TRPP) nanoplatform was constructed when the D-peptide antagonist (^D^PPA-1) of programmed death ligand-1 was conjugated onto the surface, and talabostat mesylate (Tab, a fibroblast activation protein inhibitor) was encapsulated in the watery core (^D^PPA-TRPP/Tab). Doxorubicin (DOX) conjugation in the chain served as an immunogenic cell death (ICD) inducer and hydrophobic part.

**Results:** ^D^PPA-TRPP/Tab reassembled into a micellar structure *in vivo* with TME modulation by Tab, ROS consumption by 2, 2'-diselanediylbis(ethan-1-ol), immune checkpoint blockade by ^D^PPA-1 and ICD generation by DOX. This resolved the dilemma between a hydrophilic Tab release in the TME for CAF inhibition and intracellular hydrophobic DOX release for ICD via re-assembly in weakly acidic TME with polymersome-micelle transformation. *In vivo* results indicated that ^D^PPA-TRPP/Tab could improve tumor accumulation, suppress CAF formation, downregulate regulatory T cells and promote T lymphocyte infiltration. In mice, it gave a 60% complete tumor regression ratio and a long-term immune memory response.

**Conclusion:** The study offers potential in tumor eradication via exploiting an “all-in-one” smart polymeric nanoplatform.

## Introduction

Immunogenic cell death (ICD) is characterized by a host immune response via damage-associated molecular patterns (DAMPs) (*e.g.*, calreticulin (CRT), high mobility group protein 1 (HMGB1)) and tumor-associated antigen (TAA) secretion from dying tumor cells, together facilitating dendritic cell (DC) recognition, maturation and antigen cross-presentation to T cells 1-4. Due to the efficient immune response activation, ICD induced by chemotherapeutics (*e.g.*, doxorubicin (DOX)), photosensitizers or radiosensitizers have been widely reported in cancer immunotherapies 5-8. However, as has been reported, a high level of reactive oxygen species (ROS) in the tumor microenvironment (TME) is adverse to ICD via injuring HMGB1 function 9; it is essential to consume ROS and reverse the immunosuppressive effects of the TME in order to achieve ICD of tumor cells. Moreover, immunosuppressive TME components including cancer-associated fibroblasts (CAFs), regulatory T cells (Tregs) and transforming growth factor-β (TGF-β) are also adverse to therapeutic efficacy 10-16. CAFs account for a large proportion of the TME's contribution to Treg up-regulation and TGF-β secretion 17, 18. Fibroblast activation protein (FAP) is a typical marker of CAFs, and plays a key role in fibrosis, extracellular matrix (ECM) remodeling and tumor progression 19-21. FAP may be a potential targeting point for CAFs inhibition with the aim of reversing TME mediated immunosuppression. Recently, FAP inhibitors, *e.g.*, talabostat mesylate (Tab), have been exploited for CAF inhibition with successful immunosuppressive cytokine downregulation 19.

Immune checkpoint signaling pathways, *e.g.*, programmed cell death protein 1 (PD-1)/programmed cell death ligand 1 (PD-L1), play a key role in facilitating tumors' escape from host immunosurveillance 22. Nowadays, a variety of immune checkpoint inhibitors (ICIs) including pembrolizumab, nivolumab, atezolizumab have been approved by the US Food and Drug Administration (FDA) as malignant tumor treatments 23-25. Other strategies including gene delivery to silence PD-L1 also have been reported 26. Nevertheless, the high costs of antibody or gene therapy should not be ignored. Recently, other PD-L1 inhibitors, such as D-peptide antagonist (^D^PPA-1), have been easily synthesized to an acceptable price and reported in immune checkpoint blockade studies to show potentially robust therapeutic efficacy 27-29. Therefore, exploiting synthetic antagonists of immune checkpoint proteins may be a promising strategy in exerting host immune responses to a reduced cost.

Stimulus-responsive nanomedicine is indispensable in cancer immunotherapy due to their long circulation time, high tumor accumulation and especially due to their controllable release 30-37. Stimulus (*e.g.*, redox, pH, reactive oxygen species) responsive nanosystems mainly facilitate immune responses via chemotherapeutics, photosensitizers, immune checkpoint blockade, antigens, siRNA or agonist triggered release 38-43. Furthermore, stimulus-responsive prodrug nanoplatforms, which are able to endow carrier bioactivity, reduce drug leakage and trigger drug release at the specific site, are attracting more and more attention 44, 45. Transformable stimulus-responsive nanomedicine with tunable size or morphology is in the TME capable of prolonging tumor retention and/or facilitating internalization, which reinforces its therapeutic efficacy 46-49. Therefore, transformable stimulus-responsive prodrug nanocarriers may have a huge potential in provoking host immune responses when combined with ICI and TME modulation.

**Scheme 1:**
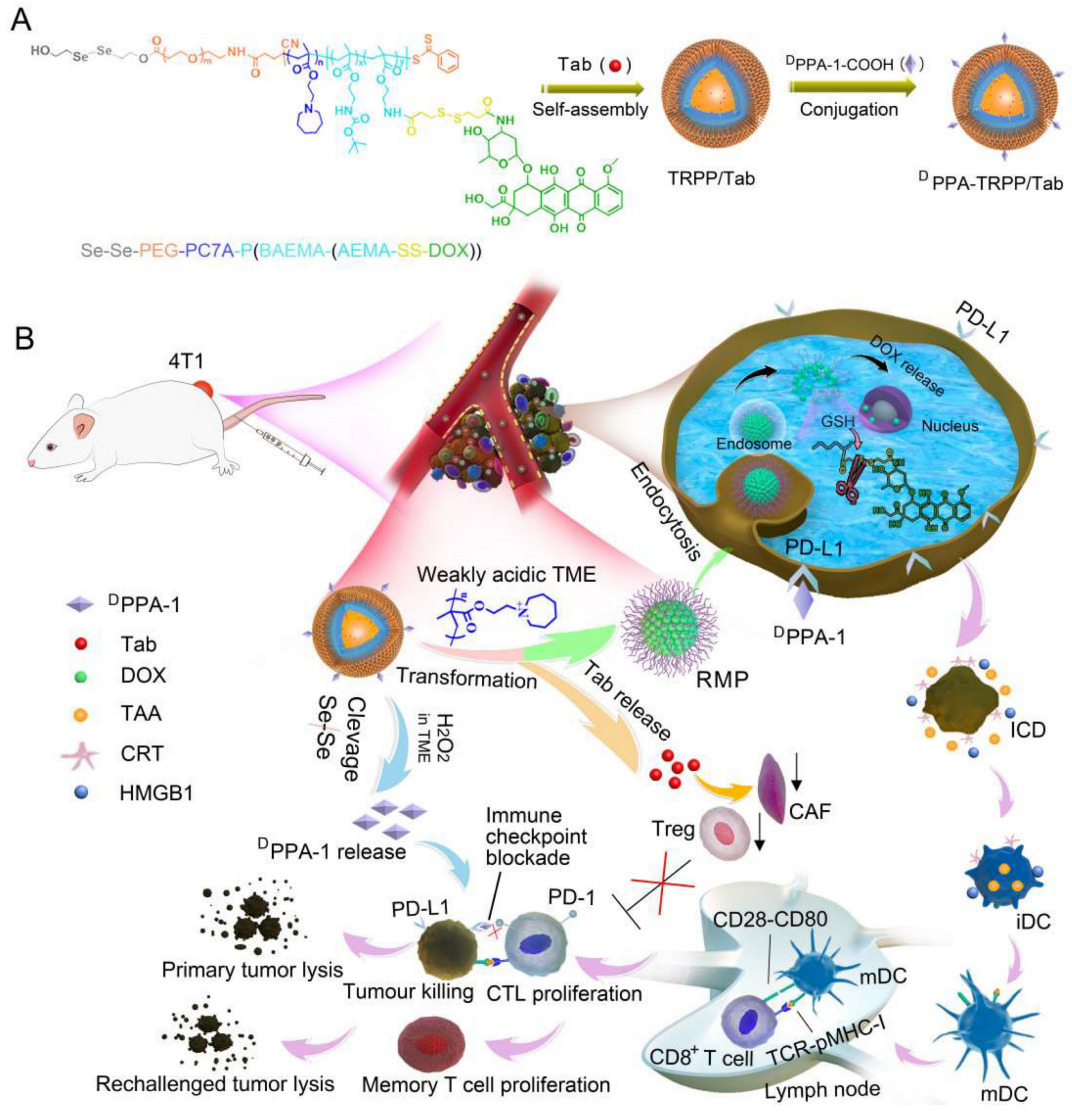
** Schematic illustration of TME and reduction dual-responsive prodrug ^D^PPA-TRPP/Tab nanoplatform for cancer immunotherapy. (A)** Preparation of ^D^PPA-TRPP/Tab. **(B)** ^D^PPA-TRPP/Tab elicits immune response *via* immune checkpoint blockade, TME modulation and immunogenic cell death.

Herein, we constructed a TME-and-reduction dual-responsive polymersomal prodrug (TRPP) nanoplatform based on copolymer 2, 2'-diselanediylbis(ethan-1-ol)-polyethylene glycol-poly 2-(hexamethyleneimino)ethyl methacrylate-poly ((2-boc-amino)ethyl methacrylate-(2-amino ethyl methacrylate-disulfide-DOX) (HO-Se-Se(dSe)-PEG-PC7A-P(BAEMA-(AEMA-SS-DOX)) with FAP inhibitor Tab encapsulation (TRPP/Tab) (**Scheme 1**). ^D^PPA-1 was able to conjugate to the TRPP/Tab surface via an esterification reaction to form ^D^PPA-TRPP/Tab. When encountering the high concentration of H_2_O_2_ in the TME, the Se-Se bond was cleaved with H_2_O_2_ consumption and ^D^PPA-1 shedding for PD-L1 blockade. The PC7A segment was converted from being hydrophobic to hydrophilic in the weakly acidic TME with re-assembly and transformation into a reduction-sensitive micellar prodrug (RMP). During re-assembly, Tab was released from TRPP into the TME for CAF inhibition. When RMP was further internalized by tumor cells, DOX was released due to the disulfide bond cleavage in the high intracellular glutathione concentration, with accompanying tumor ICD. Concurrently, ^D^PPA-TRPP/Tab inhibited Tregs, down-regulated α-smooth muscle actin (α-SMA) expression and TGF-β secretion, induced ICD, and facilitated TNF-α secretion and T lymphocyte infiltration. Remarkably, when the initial tumor volume was around 100 mm^3^, ^D^PPA-TRPP/Tab in a single dose achieved 60% complete regression in 4T1 tumor-bearing mice. The rechallenge-tumors remained notably suppressed compared with those in untreated mice due to the long-term memory immune response. For the relatively large initial tumor (~150 mm^3^), ^D^PPA-TRPP/Tab also displayed a higher antitumor activity than the other groups did. This design may provide an approach to combine multiple immunosuppressive-factors-reversal with ICD in potentiating cancer immunotherapy efficacy.

## Experimental Section

### Synthesis of pH-responsive tri-block copolymer COOH-PEG-PC7A-PBAEMA

The macromolecular RAFT reagent COOH-PEG-CPAA was obtained via amidation between COOH-PEG-NH_2_ and CPPA according to a previous report 50. The ^1^H NMR spectrum and MALDI-TOF results are presented in Figure S2-S3. (Supporting Information). HOOC-PEG-CPPA (100 mg, 0.02 mmol), MA-C7A (60 mg, 0.28 mmol) and AIBN (0.49 mg, 0.003 mmol) were dissolved in 1, 4-dioxane (2 mL) under inert atmosphere. After being stirred at 65 ºC for 24 h, the product went through precipitation, filtration and vacuum desiccation to obtain the di-block copolymer PEG-PC7A. The related characterization including ^1^H NMR and GPC results are shown in Figure S4 and Table S1 (Supporting Information). To obtain the tri-block copolymer PEG-PC7A-PBAEMA, PEG-PC7A (140 mg, 0.019 mmol), BAEMA (112 mg, 0.49 mmol) and AIBN (0.49 mg, 0.003 mmol) were dissolved in 1,4-dioxane under nitrogen protection. After being stirred at 65 ºC for 48 h, the mixture was then precipitated into a cold diethyl ether, followed by filtration. The final product (HOOC-PEG-PC7A-PBAEMA) was obtained, ^1^H NMR spectrum and GPC results of which are shown in Figure S5-S6 and Table S1 (Supporting Information).

### Synthesis and characterization of HOOC-PEG-PC7A-P(BAEMA-AEMA)

HOOC-PEG-PC7A-PBAEMA (200 mg, 0.017 mmol) and TFA (2 mL) were dissolved in DCM (2 mL). The mixture was stirred at room temperature (RT) for 0.5 h and then dialyzed against deionized water (MWCO = 3500 Da) and lyophilized. The final product (HOOC-PEG-P(BEM-BAEMA)-PC7A) was obtained as a light-yellow powder (150 mg, yield 75%). The ^1^H NMR spectrum is shown in Figure S3 (Supporting Information).

### Synthesis and characterization of dSe-PEG-PC7A-P(BAEMA-AEMA)

HOOC-PEG-PC7A-P(BAEMA-AEMA) (100 mg, 8.6 μmol) dissolved in dimethylformamide (DMF, 2 mL) was activated with EDC•HCl (2.5 mg, 13.1 μmol) and DMAP (1.6 mg, 13.1 μmol) under nitrogen atmosphere at RT for 1 h. dSe (1 mg, 12.6 μmol) was then added to the above mixture under nitrogen atmosphere. After 24 h reaction at RT, the mixture was dialyzed against deionized water (MWCO = 3.5 kDa) and lyophilized. The final product (dSe-PEG-PC7A-P(BEM-BAEMA)) was obtained as a light-yellow powder (75 mg, yield 74%) (Figure S6, Supporting Information).

### Synthesis and characterization of dSe-PEG-PC7A-P(BAEMA-(AEMA-DTPA))

3, 3-Dithiodipropionic acid (DTPA) (3.1 mg, 14.7 μmol) dissolved in DMF (2 mL) was activated with EDC•HCl (4.2 mg, 21.9 μmol) and NHS (2.5 mg, 21.9 μmol) under nitrogen atmosphere at RT for 1 h. dSe-PEG-PC7A-P(BAEMA-AEMA) (55 mg, 4.7 μmol) was dropwise added to the above mixture under nitrogen atmosphere. After being left to react for 24 h at RT, the mixture was dialyzed against deionized water (MWCO = 3.5 kDa) and lyophilized. The final product (dSe-PEG-PC7A-P(BAEMA-(AEMA-DTPA) was obtained as a light-yellow powder (50 mg) (Figure S7, Supporting Information).

### Synthesis and characterization of dSe-PEG-PC7A-P(BAEMA-(AEMA-SS-DOX)

dSe-PEG-PC7A-P(BAEMA-(AEMA-DTPA)) (40 mg, 3.33 μmol) dissolved in DMF (2 mL) was activated with EDC•HCl (2.9 mg, 15 μmol) and NHS (1.7 mg, 15 μmol) under nitrogen atmosphere at RT for 1 h. DOX (5.8 mg, 10 μmol) was then added to the above mixture under nitrogen atmosphere. After being left to react for 24h at RT, the mixture was dialyzed against deionized water (MWCO = 3.5 kDa) and lyophilized. dSe-PEG-PC7A-P(BAEMA-(AEMA-SS-DOX)) was obtained as a red powder, the DOX content in which was characterized by UV-vis (Figure S8, Supporting Information).

### DOX conjugation ratio detection

Free DOX, with a series of different concentrations from low to high, 5, 10, 15, 20, 25, 30, 35, 40 μg/mL, was measured by UV-vis for the standard curve. Pro-drug copolymer dSe-PEG-PC7A-P(BAEMA-(AEMA-SS-DOX)) dissolved in DMF (1 mg/mL) was detected by UV-vis to obtain the absorbance curve. The DOX content in each polymer chain was calculated according to the absorption intensity and standard curve.

### H_2_O_2_ consumption investigation

Prodrug copolymer dSe-PEG-PC7A-P(BAEMA-(AEMA-SS-DOX)) was dissolved in DMF to prepare different concentration solutions (1 mg/mL, 5 mg/mL) with H_2_O_2_ (100 μM) addition. A H_2_O_2_ fluorescence probe (Oxi Vision green^TM^) was added to the above solutions, and to a H_2_O_2_ standard solution, then all were incubated for 1 h at RT. The H_2_O_2_ concentration changes in each sample were detected via fluorescence spectroscopy.

### Preparation of TME, pH and reduction triple-responsive polymersomal prodrug nanoplatform (TRPP)

The TRPP was fabricated by a solvent-exchange method. Prodrug copolymer dSe-PEG-PC7A-P(BAEMA-(AEMA-SS-DOX)) dissolved in THF (200 μL, 5 mg/mL) was dropwise added into phosphate buffered solution (PBS, 800 μL, 10 mM, pH 7.4) to acquire the uniform nanoplatform. A TME responsive polymersome (TP), as a control, was prepared by a similar method, it self-assembled from copolymer PEG-PC7A-PBAEMA. The dynamic sizes and morphology were separately measured by dynamic latter scattering (DLS) and by transmission electron microscopy (TEM).

### Preparation of^ D^PPA-1 decorated TRPP (^D^PPA-TRPP)

^D^PPA-1 was able to conjugate on the TRPP surface to form ^D^PPA-TRPP. In detail, ^D^PPA-1 (55 μg, 0.026 μmol) first reacted with EDC.HCl (13.6 μg, 0.071 μmol), DMAP (8.6 μg, 0.071 μmol) and triethylamine (TEA, 10 μL, 0.071 μmol) for 30 min at 30 ºC. The reactants were then added dropwise into TRPP (1 mg/mL, 1 mL) and stirred overnight. After dialysis (MWCO, 3500), ^D^PPA-TRPP was obtained, the size and size distribution of which were measured by DLS. The morphology was characterized by TEM.

### ^D^PPA-TRPP transformation into micellular nanoplatform investigation

^D^PPA-TRPP transformation was explored by size and morphology changes, respectively. ^D^PPA-TRPP at pH 6.8 or 7.4 was monitored by DLS at pre-set time points to show the pH responsiveness and re-assembly. Morphologies were captured by high angle annular dark field-scanning transmission electron microscopy (HAADF-STEM) at 0 and 24 h (pH 6.8) for further transformation confirmation. One drop of ^D^PPA-TRPP was put on carbon-coated copper grids and air-dried overnight at RT before capturing images.

### Animals and cells

BALB/c mice (female, 6-8 weeks, 20 g) were purchased from SPF (Beijing) Biotechnology Co., Ltd. All the animal experiments were executed with ethical compliance and following the protocol from the Institute of Drug Discovery & Development of Zhengzhou University (syxk (yu) 2018-0004). 4T1 cells were cultured in Dulbecco's modified eagle media (DMEM) containing 10% fetal bovine serum (FBS) and 1% penicillin & streptomycin (P&S) (37 ºC, 5% CO_2_).

### *In vivo* antitumor activity investigation

BALB/c mice were inoculated with 4T1 cells (2.0 × 10^6^ per mouse) in the right flank. Five formulations, PBS, TRPP, ^D^PPA-TRPP, TRPP/Tab, ^D^PPA-TRPP/Tab (^D^PPA: 0.5 mg/kg, DOX: 0.5 mg/kg, Tab: 1.0 mg/kg), were used to treat the tumor-bearing mice. As for the small initial tumor volume treatment, drug formulations were given at day-6 post-inoculation when tumor volumes were around 100 mm^3^. Tumor volume and body weight in each group were measured every three days. The formula used to calculate tumor volume was V = 0.5 * Length * Width^2^. Tumor volume recordings were stopped when the volumes reached 1500 mm^3^ at day-27. The survival of mice was observed and recorded within 45 days. The surviving mice were rechallenged with 4T1 cells for the memory immune response study when the untreated mice were also inoculated. The rechallenge-tumor volume was monitored and measured every three days with untreated mice as a control. Mice were euthanatized when the tumor volume in the untreated mice was around 1500 mm^3^ at day 72. Tumors from each group were photographed and weighed.

As for the mice with larger initial tumor volume (~150 mm^3^), different formulations were intravenously administered at day 9 post-inoculation. Tumor volume and body weight were measured until the tumor volume reached 1500 mm^3^. Mice were euthanatized at the therapeutic endpoint, at which tumor and major organs, *e.g.*, heart, liver, spleen, lung and kidneys, were extracted for H&E staining. Apoptosis of tumor tissues was also evaluated by terminal-deoxynucleotidyl transferase mediated nick end labeling (TUNEL) assay.

### Cytokine detection

HMGB1 from the supernatant in 4T1 cells treated by TRPP was measured via ELISA according to the manufacturer's protocol. IL-12, TNF-α and TGF-β levels in serum were also detected via ELISA according to standard protocols.

### Flow cytometry measurement for T cell populations

Tumors, after extraction from treated mice, were cut into small pieces, mechanically grinded and then digested by collagenase (50 U/mL), hyaluronidase (100 μg/mL) and DNAse (50 U/mL) for 2 h at 37 ºC. Cells then underwent filtration, centrifugation and washing by PBS. Cells were stained with anti-CD3e-PE, anti-CD8a-APC, and anti-CD4-Percp/Cy5.5 for CD8^+^CD4^+^ T cells, anti-CD4-Percp/Cy5.5 and anti-Foxp3-FITC for Tregs for 30 min at RT. After further washing and centrifugation, cells were suspended in PBS and analyzed by flow cytometry.

### Immunofluorescence staining assay

Extracted tumors from mice were embedded with Tissue-Tek O.C.T. Compound, frozen at -80 ºC for 48 h. Tumor sections were obtained using cryotomy, and stained with different primary antibodies. Anti-CD31 and anti-α-SMA were used for CAF characterization. Anti-CD3, anti-CD8 and anti-CD4 were used for CD8^+^ and CD4^+^ T cell infiltration investigation. Anti-CRT was exploited for ICD studies. The above primary antibodies were stained overnight at 4 ºC, followed by fluorescence-labeled secondary antibodies (Goat anti-Rat Alexa Fluor^®^ 488, Donkey anti-Rabbit Alexa Fluor^®^ 594 staining. The slides were captured by confocal microscopy (CLSM).

### Statistical analysis

Data was expressed as mean ± standard deviation (SD) of at least three independent experiments. The numbers of samples per group (n) are specified in the figure legends. Comparison of parameters for more than three groups were performed by one-way analysis of variance (ANOVA) followed by Tukey's significant difference post-hoc test. All statistical analyses were conducted using GraphPad Prism 7. P-values less than 0.05 were considered statistically significant (^*^P < 0.05, ^**^P < 0.01, ^***^P < 0.001, ^****^P < 0.0001, or NS > 0.05).

## Results and Discussion

### Preparation and Characterization of TRPP

To construct the TME and reduction dual responsive nanoplatform, we first synthesized a pH responsive monomer MA-C7A via a substitution reaction (Scheme S1) and the macro reversible addition-fragmentation chain transfer (RAFT) agent PEG-CPPA was obtained via amidation reaction (Scheme S2). Figure S1 showed that the pure monomer MA-C7A was obtained. As shown in Figure S2, the conversion ratio of CPPA was as high as 94.6%, read from its ^1^H NMR spectrum. The MALDI-TOF spectra in Figure S3 also indicated the successful synthesis of PEG-CPPA. The pH sensitive di-block PEG-PC7A and tri-block copolymer COOH-PEG-PC7A-PBAEMA were synthesized via a reversible addition-fragmentation chain transfer (RAFT) polymerization (Scheme S3). As shown in Figure S4 and Figure S5, the molecular weights of PEG-PC7A and COOH-PEG-PC7A-PBAEMA were 5.0-2.3 and 5.0-2.3-4.7 kg/mol, respectively, according to the ^1^H NMR spectra. The relative molecular weights of the two block copolymers were 9.3 (di-block, *M*_w_/*M*_n_: 1.22) and 11.8 kg/mol (tri-block, *M*_w_/*M*_n_: 1.24), respectively, from gel permeation chromatography (GPC) results, in agreement with the ^1^H NMR spectra (Figure S6, Table S1). After hydrolysis, COOH-PEG-PC7A-P(BAEMA-AEMA(NH_2_)) was obtained (Scheme S4); the hydrolysis degree was 13.6% according to the ^1^H NMR result in Figure S7. The primary amine (NH_2_) content was 2.7 NH_2_ in each polymer chain detected by a 2, 4, 6-trinitrobenzene sulfonic acid (TNBSA) assay (Figure S8), which was consistent with the ^1^H NMR result (Figure S7).

**Figure 1:**
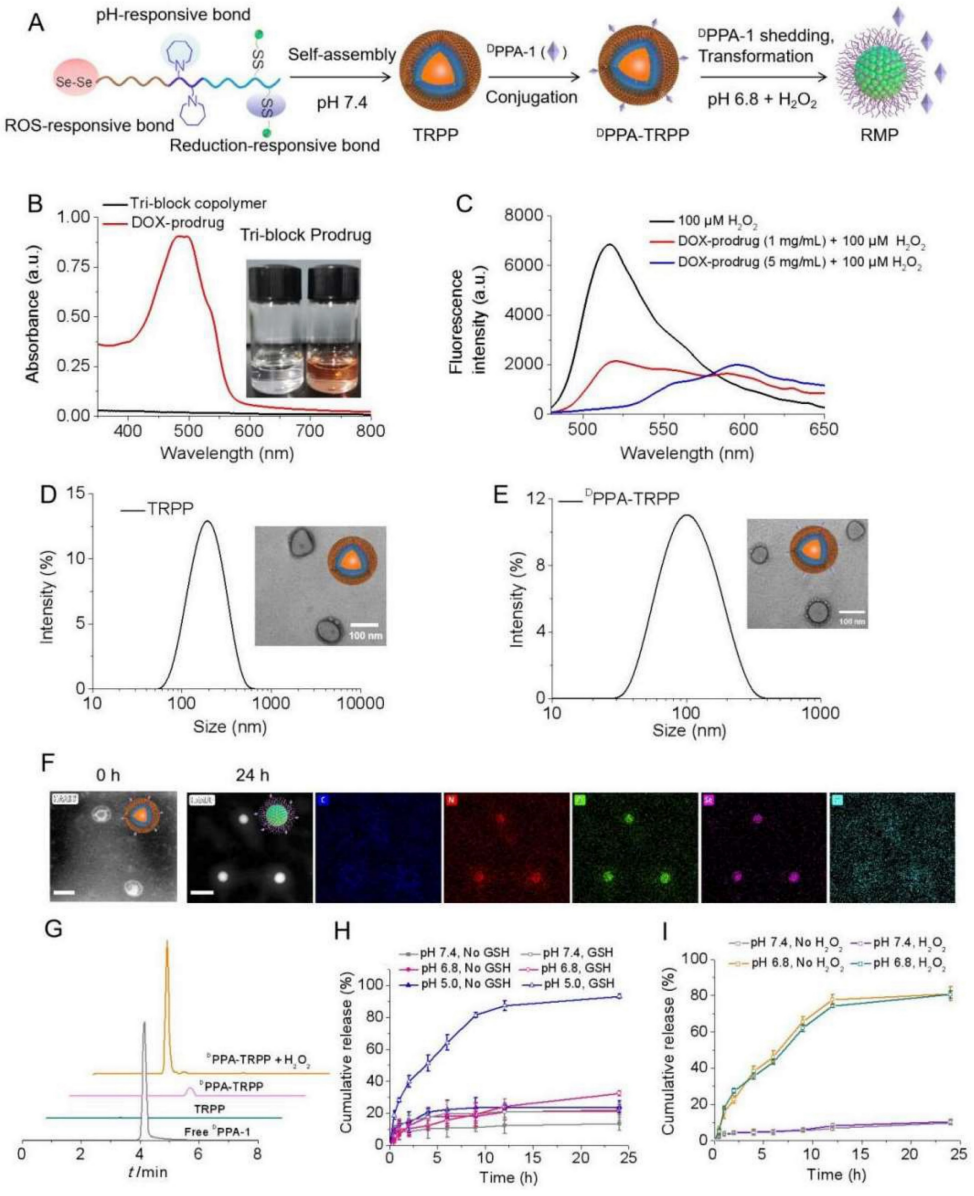
***In vitro* construction and characterization of TME and reduction dual responsive polymersomal prodrug (TRPP) nanoplatform and ^D^PPA-1 decorated TRPP (^D^PPA-TRPP). (A)** Schematic illustration of preparation, pH and ROS responsiveness of ^D^PPA-TRPP. **(B)** UV-vis spectrum of prodrug copolymer. Inset image presented tri-block or prodrug copolymer. **(C)** *In vitro* H_2_O_2_ (100 µM) consumption mediated by prodrug copolymer. **(D)** Size and morphology of TRPP measured by DLS and TEM, respectively. **(E)** ^D^PPA-TRPP characterization by DLS and TEM. **(F)** Morphology transformation of ^D^PPA-TRPP from polymersomes into micelles at pH 6.8 characterized by HAADF-STEM. Result at 0h represented the HAADF-STEM image of ^D^PPA-TRPP. Result at 24h represented the STEM-EDC mapping images of ^D^PPA-RMP. **(G)** ^D^PPA-1 shedding from ^D^PPA-TRPP in the condition of H_2_O_2_ (100 µM) detected by HPLC. **(H)** *In vitro* DOX release from ^D^PPA-TRPP under different conditions within 24 h measured by fluorescence spectrophotometer. (n = 3, mean ± SD). **(I)** *In vitro* Tab release from ^D^PPA-TRPP under different conditions within 24 h detected by LC-MS (n = 3, mean ± SD).

2, 2'-Diselanediylbis(ethan-1-ol) (HO-Se-Se-OH, written as dSe) was obtained after a substitution reaction (Scheme S5), with satisfactory purity as read from the ^1^H NMR and ^13^C NMR spectra in Figure S9. According to Figure S10 (Supporting Information) for the Fourier-transform infrared spectroscopy (FT-IR) characterization, Se-Se bond cleavage was notably observed with seleninic acid formation in the presence of H_2_O_2_ (100 µM) based on the characteristic absorption band at 880 cm^-1^ indicating the ROS consumption of dSe. H_2_O_2_ and pH dual responsive HO-Se-Se(dSe)-PEG-PC7A-P(BAEMA-AEMA) was acquired after an esterification reaction (Scheme S6), which was characterized by ^1^H NMR and infrared spectra as shown in Figure S11. DTPA was able to react with dSe-PEG-PC7A-P(BAEMA-AEMA) via an amidation reaction (Scheme S7), Figure S12 shows the successful conjugation. After amidation with DOX, the TME and reduction dual responsive prodrug (dSe)-PEG-PC7A-P(BAEMA-(AEMA-SS-DOX)) was obtained (Scheme S8, Figure 1A). The schematic illustration for preparation and pH responsiveness of TRPP is shown in Figure 1A. According to Figure 1B and Figure S13, DOX mass fraction in the prodrug copolymer was 5% as detected by UV-vis, with the average number of DOX being 1.2 in each prodrug copolymer chain. The orange color of the prodrug copolymer also demonstrated successful DOX conjugation. H_2_O_2_ responsiveness of the prodrug copolymer was subsequently investigated using a TME mimicking ROS concentration. As shown in Figure 1C, the H_2_O_2_ (100 µM) amount significantly decreased with an attenuated peak absorption at 520 nm after prodrug treatment. With increasing prodrug concentration, the H_2_O_2_ consumption increased (Figure 1C).

The prodrug copolymer could self-assemble into a TRPP nanoplatform via a solvent-exchange method. According to the DLS result, TRPP had a hydrodynamic size of 174 ± 4 nm with a narrow distribution (0.13 ± 0.02) (Figure 1D). The morphology was characterized by TEM, which confirmed its hollow structure (Figure 1D). The TP was fabricated by PEG-PC7A-PBAEMA as a control, the size of which measured by DLS was 140 ± 2 nm with narrow distribution (0.11 ± 0.01) (Figure S14). ^D^PPA-1 was able to conjugate onto the TRPP surface via an esterification reaction to form ^D^PPA-TRPP (Figure 1A). The DLS result indicated that ^D^PPA-TRPP had a hydrodynamic size of 134 ± 5 nm with a relatively narrow distribution (0.19 ± 0.05), the hollow morphology of which was confirmed by TEM (Figure 1E). As shown in Figure S15, ^D^PPA-TRPP increased at 8 h under weakly acidic condition (pH 6.8), while it re-assembled into smaller ones at 24 h, indicating its pH responsiveness. In contrast, a negligible size change was observed when ^D^PPA-TRPP was placed in the neutral PBS (pH 7.4, 10 mM, 150 mM NaCl), showing its stability under physiological conditions. As shown in Figure 1F, a clear hollow structure of ^D^PPA-TRPP was observed from the HAADF-STEM image. The noticeable micellar transformation of ^D^PPA-TRPP was seen at pH 6.8 at 24 h when densities of elements C, N, O Se and S were observed on the particle surface from STEM-EDC mapping results (Figure 1F). We speculated that the morphological transformation may be attributed to the PC7A segment changing from hydrophobic to hydrophilic at a low pH value. As shown in Figure S15B-D, ^D^PPA-TRPP had a superior stability in PBS, blood and cell culture medium, with negligible size changes.

^D^PPA-1 was able to cleave from TRPP in the presence of H_2_O_2_ (100 µM) (Figure 1G), measured by High Performance Liquid Chromatography (HPLC). As shown in Figure 1H, DOX was rapidly released from ^D^PPA-TRPP with a cumulative release as high as 93.1% in a pH 5.0 10 mM GSH solution within 24 h. Without GSH, or with 10 mM GSH at pH 7.4, DOX release was in the range of 21.0-22.1%. At pH 6.8, with 10 mM GSH, the cumulative release was slightly higher (32.5%) than in the other control groups. The above results indicated that both low pH and high reduction conditions are essential for facilitating DOX release. Tab could be encapsulated in both TRPP and ^D^PPA-TRPP to form TRPP/Tab and ^D^PPA-TRPP/Tab, respectively. As shown in Table S2, both TRPP and ^D^PPA-TRPP were able to efficiently encapsulate Tab when the drug loading efficiency (DLE) was high as 81.3-84.8% in a theoretical drug loading content (DLC) as 5%. The highest DLC reached 8.57% when the size changed from 118 ± 3 to 132 ± 4 nm, with a relatively narrow PDI (Table S2). Tab was rapidly released from ^D^PPA-TRPP at pH 6.8, with or without H_2_O_2_, the cumulative release was as high as 80.9% within 24 h (Figure 1I). In contrast, at pH 7.4, the release was around 10%.

### *In vitro* cellular behavior and* in vivo* NIR imaging

**Figure 2:**
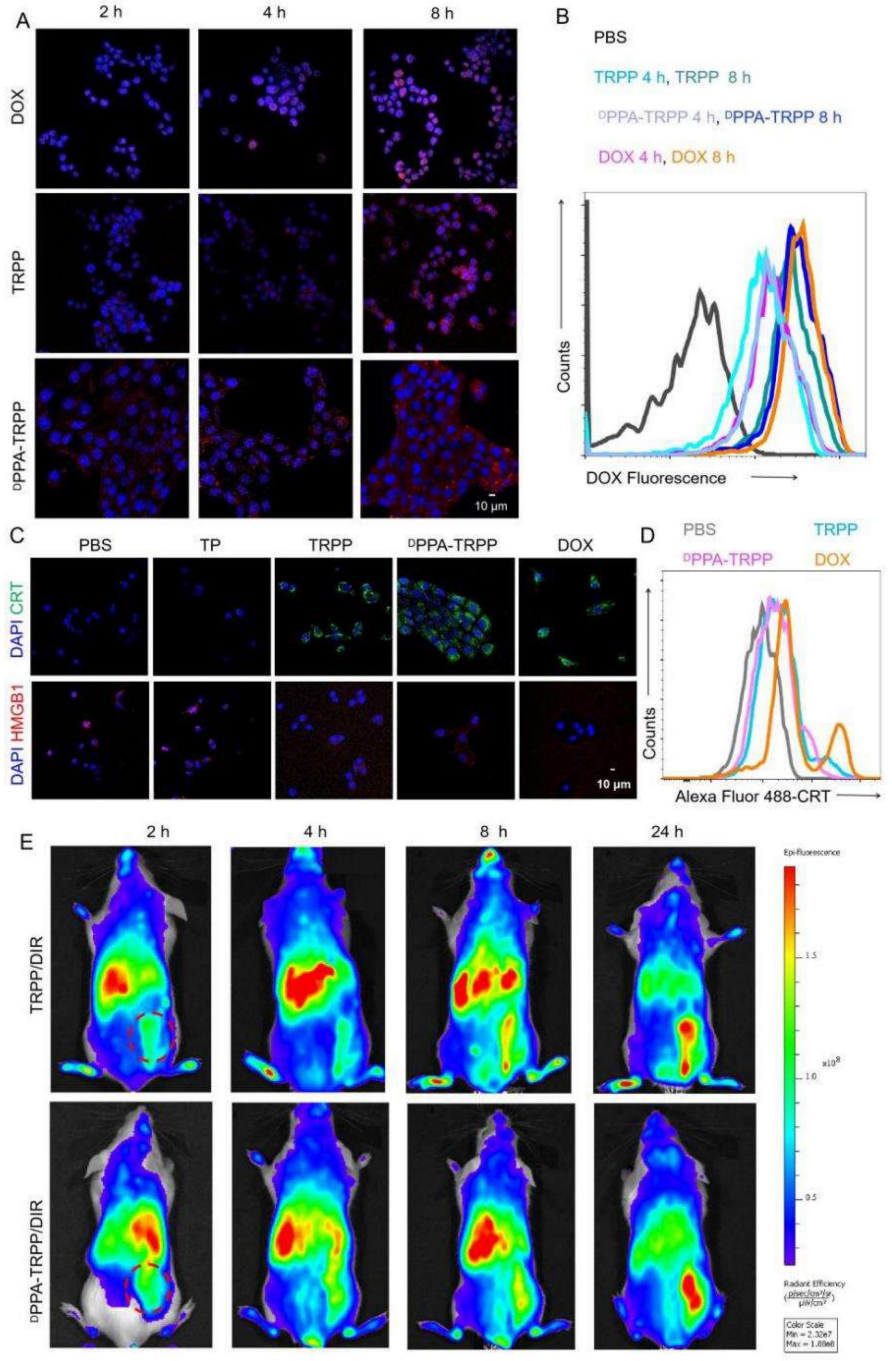
**Cellular behavior and *in vivo* tumor accumulation investigation. (A)** Cellular internalization of ^D^PPA-TRPP in 4T1 cells characterized by CLSM and **(B)** flow cytometry, respectively. **(C)** ^D^PPA-TRPP mediated ICD via CRT exposure and HMGB1 release via CLSM characterization. Blue color presented DAPI. Green and red colors were CRT and HMGB1, respectively. **(D)** TRPP induced ICD by flow cytometry characterization. **(E)*** In vivo* tumor accumulation of ^D^PPA-TRPP/DIR and TRPP/DIR at different time points.

The cellular behavior of ^D^PPA-TRPP was subsequently investigated. TRPP induced potent cytotoxicity in 4T1 cells due to DOX conjugation with an IC_50_ of 1.39 µg/mL, which is similar to that of free DOX (1.25 µg/mL) (Figure S16, Supporting Information). As shown in Figure 2A and B, ^D^PPA-TRPP could be rapidly internalized by 4T1 cells with the red signal (DOX) observed with CLSM (Figure S17) and in the form of a noticeable fluorescence shift during flow cytometry. ^D^PPA-TRPP induced ICD in 4T1 cells when CRT exposure and HMGB1 release were observed from CLSM (Figure 2C, Figure S18) and ELISA (Figure S19). Less HMGB1 release was observed in TP treated cells, indicating that DOX conjugation triggered ICD. Flow cytometry results in Figure 2D with a notable CRT fluorescence shift further confirmed ^D^PPA-TRPP mediated ICD. In addition, whether the nanocarrier TP itself without DOX conjugation induces immunity is an interesting point. As seen in Figure S20, TP could induce DC maturation with a 19.0 ± 1.6% CD80^+^CD86^+^ ratio in CD11c^+^ DC 2.4 cells, which was 1.7-fold higher than that of PBS (11.5 ± 0.9%). In addition, TRPP and ^D^PPA-TRPP mediated ICD with DAMPs release also facilitated DC maturation with 26.5 ± 1.3% and 27.5 ± 1.3% CD80^+^CD86^+^ DCs detected, which was approximately 2.5-fold higher than that of PBS (10.7 ± 1.2%, Figure S21).

We then investigated *in vivo* tumor accumulation of ^D^PPA-TRPP, which was the precondition for successful TME modulation and antitumor activity enhancement. To conveniently monitor tumor accumulation at different time points, Tab was replaced by lipophilic DIR as the fluorescent model compound encapsulation in ^D^PPA-TRPP to form ^D^PPA-TRPP/DIR. As shown in Figure 2E, the fluorescent signal from tumor tissue increased over time in mice with TRPP/DIR or ^D^PPA-TRPP/DIR treatment within 24 h, indicating the prolonged retention time of this nanoformulation.

### *In vivo* TME modulation investigation

**Figure 3:**
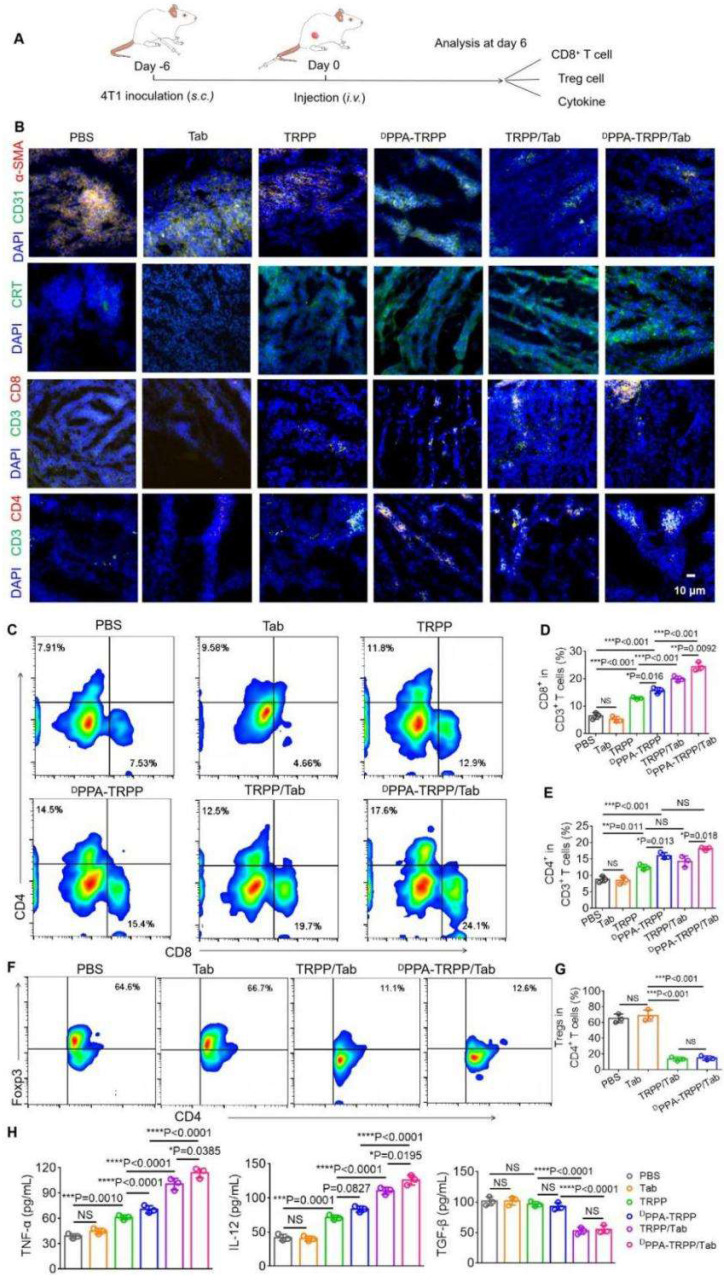
***In vivo* tumor microenvironment modulation after ^D^PPA-TRPP/Tab treatment at day 6 after 4T1 tumor inoculation into BALB/c mice.** The mice were divided into PBS, Tab, TRPP, ^D^PPA-TRPP, TRPP/Tab, and ^D^PPA-TRPP/Tab groups (n = 3/group). **(A)** Schematic illustration for the tumor inoculation, drug administration and analysis. **(B)** α-SMA staining for CAFs characterization, CRT staining for ICD, CD8^+^ and CD4^+^ T cell infiltration in tumor tissue after different treatments. **(C)** Representative CD8^+^ CD4^+^ T cell percentages and **(D, E)** quantitative analysis in tumor tissue after different treatments by flow cytometry measurement. **(F)** Representative Foxp3^+^ CD4^+^ percentage and **(G)** quantitative analysis. **(H)** Cytokines TNF-α, IL-12 and TGF-β in serum were detected via ELISA after treatment. ^*^P < 0.05, ^**^P < 0.01, ^***^P < 0.001, ^****^P < 0.0001 by analysis of ANOVA with Tukey's post-hoc test.

The nanosystem mediated TME modulation was subsequently explored (Figure 3A). As shown in Figure 3B, weak α-SMA signals (red color) were observed in tumor tissue for mice with ^D^PPA-TRPP/Tab and TRPP/Tab treatments, illustrating CAF inhibition (Figure S22a). Notable α-SMA fluorescence existed in mice treated by PBS, free Tab, TRPP, and ^D^PPA-TRPP, showing that Tab encapsulation in this nanosystem induced TME modulation (Figure 3B, Figure S22a). Notable CRT exposure (green color) was observed in all nanoformulated treatment groups, but not in single Tab treated mice, indicating DOX conjugation mediated ICD (Figure 3B, Figure S22b). CRT expression was not affected by ^D^PPA-1 attachment and Tab encapsulation in TRPP nanoformulations, indicating that the DOX conjugation induced ICD. Impressively, ^D^PPA-TRPP/Tab treated mice had the most CD8^+^ and CD4^+^ T cell infiltration compared with the other groups, mainly attributed to the combination of ^D^PPA-1 mediated immune checkpoint blockade, Tab-induced TME modulation, and DOX-caused ICD (Figure 3B, Figure S23). Flow results shown in Figure 3C-G were similar to those in Figure 3B with CD8^+^, CD4^+^ T cell percentage increments and CD4^+^ Foxp3^+^ T cell percentage decrements for nanoformulation treated groups. In short, mice given ^D^PPA-TRPP/Tab treatments had the highest CD8^+^CD4^+^ percentage (42.7 ± 1.5%) compared with the other groups (PBS: 15.3 ± 1.0%, Tab: 13.7 ± 0.5%, TRPP: 25.5 ± 1.0%, ^D^PPA-TRPP: 31.7 ± 1.6%, TRPP/Tab: 34.2 ± 1.5%) (Figure 3D, E). The Foxp3^+^CD4^+^ ratio was significantly decreased in mice given the Tab nanoformulation treatment (14.3 ± 2.2%) compared with mice given PBS (65.4 ± 4.6%) or Tab (68.9 ± 5.8%) (Figure 3G). Mice treated with Tab nanoformulations had the highest TNF-α and IL-12 levels compared with the other groups (Figure 3H). TGF-β was as expected suppressed in Tab nanoformulation treated mice compared with those receiving PBS, single Tab, TRPP or ^D^PPA-TRPP treatments (Figure 3H). The results suggest that TME modulation mediated by Tab, ICD induced by DOX and PD-1/PD-L1 blockade by ^D^PPA-1 together facilitated CD8^+^ CD4^+^ T cell infiltration for a potent immune response.

### *In vivo* antitumor immunity and long-term memory immune response

**Figure 4:**
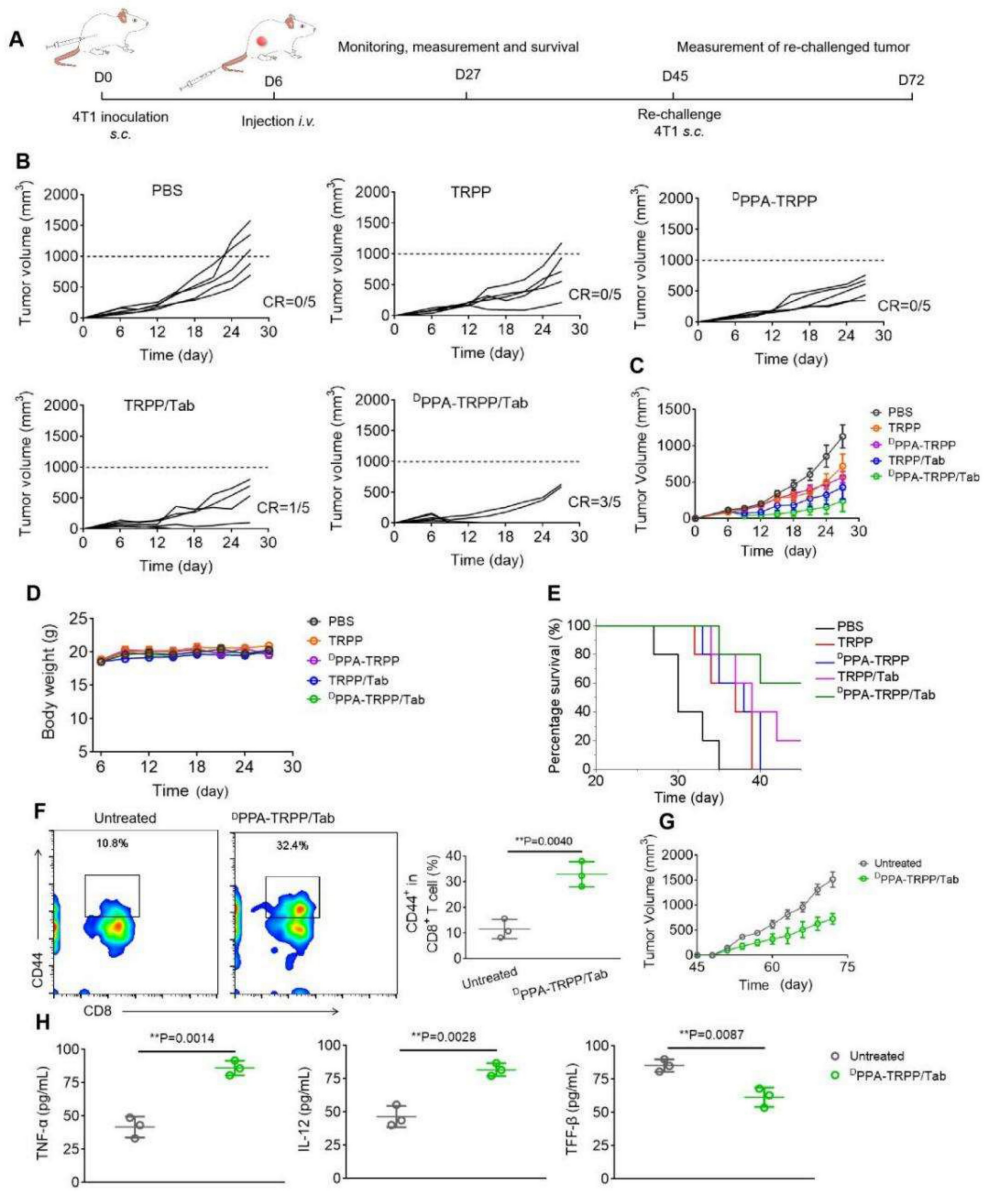
***In vivo* antitumor activity and long-term memory immune response of ^D^PPA-TRPP/Tab in 4T1 tumor model. (A)** Schematic illustration for the tumor inoculation, drug administration, tumor volume monitoring and analysis. The drug was administered at day 6 post inoculation. Mice were randomly divided into five groups: PBS, TRPP, ^D^PPA-TRPP, TRPP/Tab, and ^D^PPA-TRPP/Tab (n = 5/group). **(B)** Individual and **(C)** average tumor volume growth curve after different treatments. CR represented complete tumor regression. **(D)** Body weight changes. **(E)** Survival curves. **(F)** CD44^+^ CD8^+^ T cell percentage and quantitative analysis. **(G)** Rechallenge-tumor volume growth curve. **(H)** Cytokine detection in serum of mice at day 7 post rechallenging. ^**^P < 0.01 by analysis of ANOVA with Tukey's post-hoc test.

Since the ultimate purpose of devising the project was tumor growth inhibition, the *in vivo* antitumor activity of ^D^PPA-TRPP/Tab was then investigated in 4T1 tumor-bearing BALB/c mice. The mice were randomly divided into five groups of PBS, TRPP, ^D^PPA-TRPP, TRPP/Tab and ^D^PPA-TRPP/Tab, at day 6 after tumor inoculation when their tumor volumes were approximately 100 mm^3^. The schematic illustration for tumor inoculation, drug administration, observation, tumor re-challenging and analysis is shown in Figure 4A. As shown in Figure 4B and C, the tumor volume and growth were significantly suppressed in mice undergoing ^D^PPA-TRPP/Tab treatment, with a complete tumor regression (CR) ratio as high as 60%. The CR ratio in TRPP/Tab treated mice was 20% (Figure 4B and C). The ^D^PPA-TRPP treated group showed slightly higher antitumor activity than did the TRPP-only treated group, both treatments were superior to PBS. The tumor images of treated mice at day 27 after inoculation were shown in Figure S24, these also confirmed the highest antitumor effect of ^D^PPA-TRPP/Tab. Negligible body weight changes were observed in mice after treatment, indicating that the treatments were well tolerated (Figure 4D). ^D^PPA-TRPP/Tab treated mice had prolonged survival time compared with mice in the other groups (Figure 4E).

Mice with complete tumor regression were rechallenged at day 45 post first tumor inoculation, with the untreated mice serving as controls. Before the re-challenging, memory T cells were analyzed from plasma. As shown in Figure 4F, mice subjected to the ^D^PPA-TRPP/Tab treatment had a higher percentage of CD8^+^CD44^+^ T cells (32.9 ± 4.0%) than untreated mice (11.6 ± 3.1%). As shown in Figure 4G, the rechallenge-tumors were notably suppressed in mice undergoing the ^D^PPA-TRPP/Tab treatment compared with the tumors in untreated mice. Tumor images and weight at day 72 post inoculation further confirmed the high antitumor activity of ^D^PPA-TRPP/Tab (Figure S25). Negligible body weight loss was observed in mice with rechallenge-tumors (Figure S26). As shown in Figure 4H, higher TNF-α and IL-12 levels, and lower TGF-β concentrations were detected in mice undergoing ^D^PPA-TRPP/Tab treatment than in untreated mice. The above results indicated a superior antitumor activity of ^D^PPA-TRPP/Tab even in a single dose, with indicated high CR ratio, long survival time and robust tumor growth inhibition even for tumor re-challenged mice.

### *In vivo* antitumor and further mechanism investigation for larger initial tumor

**Figure 5:**
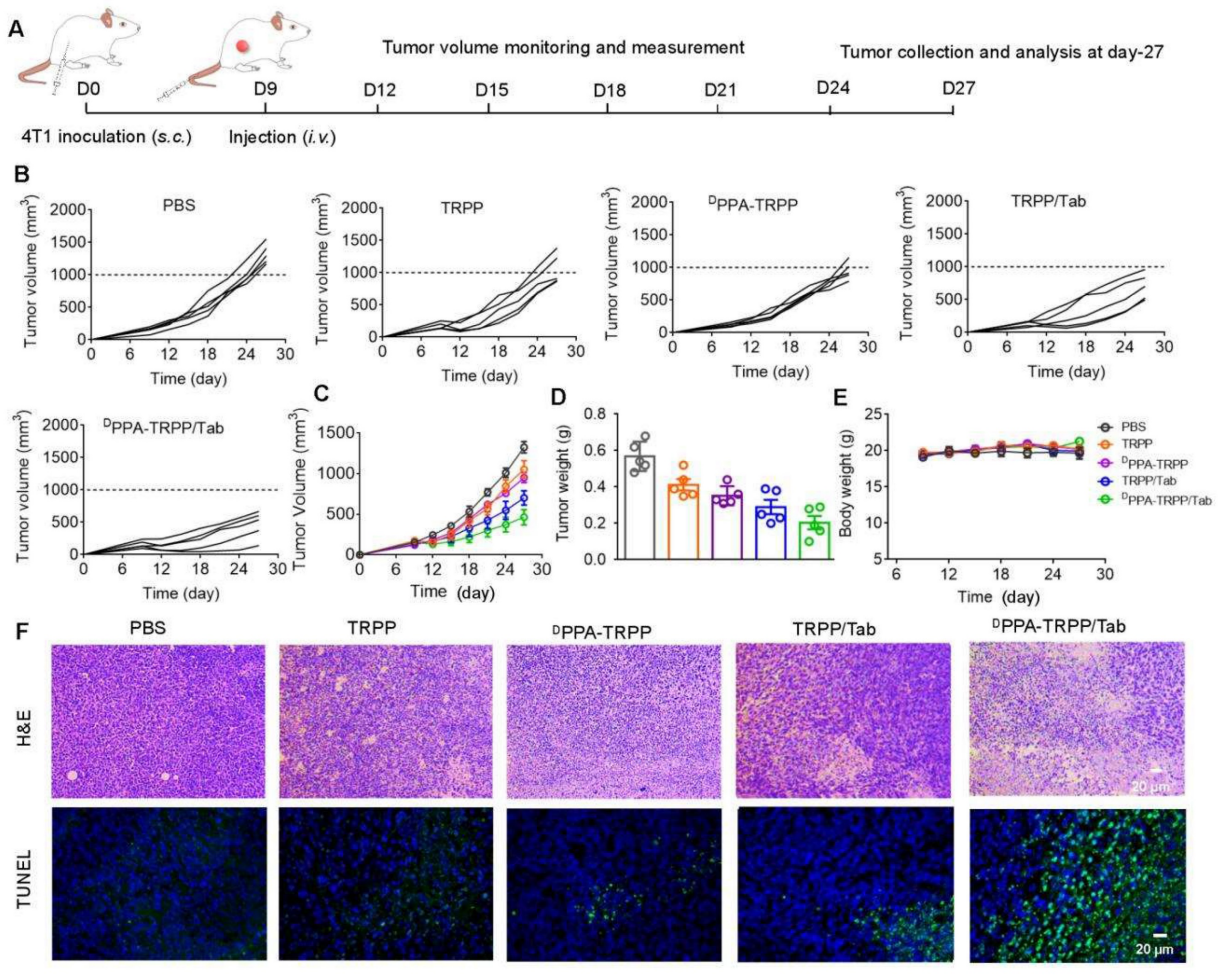
***In vivo* antitumor activity of ^D^PPA-TRPP/Tab in 4T1 tumor model. (A)** Schematic illustration for the tumor inoculation, drug administration, tumor volume monitoring and analysis. Drug formulations were administered at day 9 post-inoculation. Mice were randomly divided into five groups: PBS, TRPP, ^D^PPA-TRPP, TRPP/Tab, and ^D^PPA-TRPP/Tab (n = 5/group). **(B)** Individual and **(C)** average tumor volume growth curves after treatment. **(D)** Tumor weight at the therapeutic endpoint. **(E)** Body weight changes. **(F)** H&E staining and TUNEL results of tumor tissues.

Inspired by the robust antitumor activity of ^D^PPA-TRPP/Tab for mice with relatively small initial tumor volume (~100 mm^3^), we then investigated the antitumor efficacy against a larger initial tumor (~150 mm^3^). The detailed schematic illustration is shown in Figure 5A. According to Figure 5B and C, even though no complete tumor regression was observed, mice undergoing the ^D^PPA-TRPP/Tab treatment had the best antitumor activity compared with PBS, TRPP, ^D^PPA-TRPP and TRPP/Tab treated mice. Mice treated with ^D^PPA-TRPP/Tab had the lowest tumor volume compared with the mice in other groups (Figure 5D), which was confirmed by tumor photographs in Figure S27. No obvious body weight changes (Figure 5E) and negligible normal organ damages (Figure S28) were observed in all groups showing again that the treatments were well tolerated. The noticeable cell death was observed in mice undergoing the ^D^PPA-TRPP/Tab treatment from hematoxylin and eosin (H&E) staining and TUNEL results indicating its potent tumor lethality (Figure 5F).

**Figure 6:**
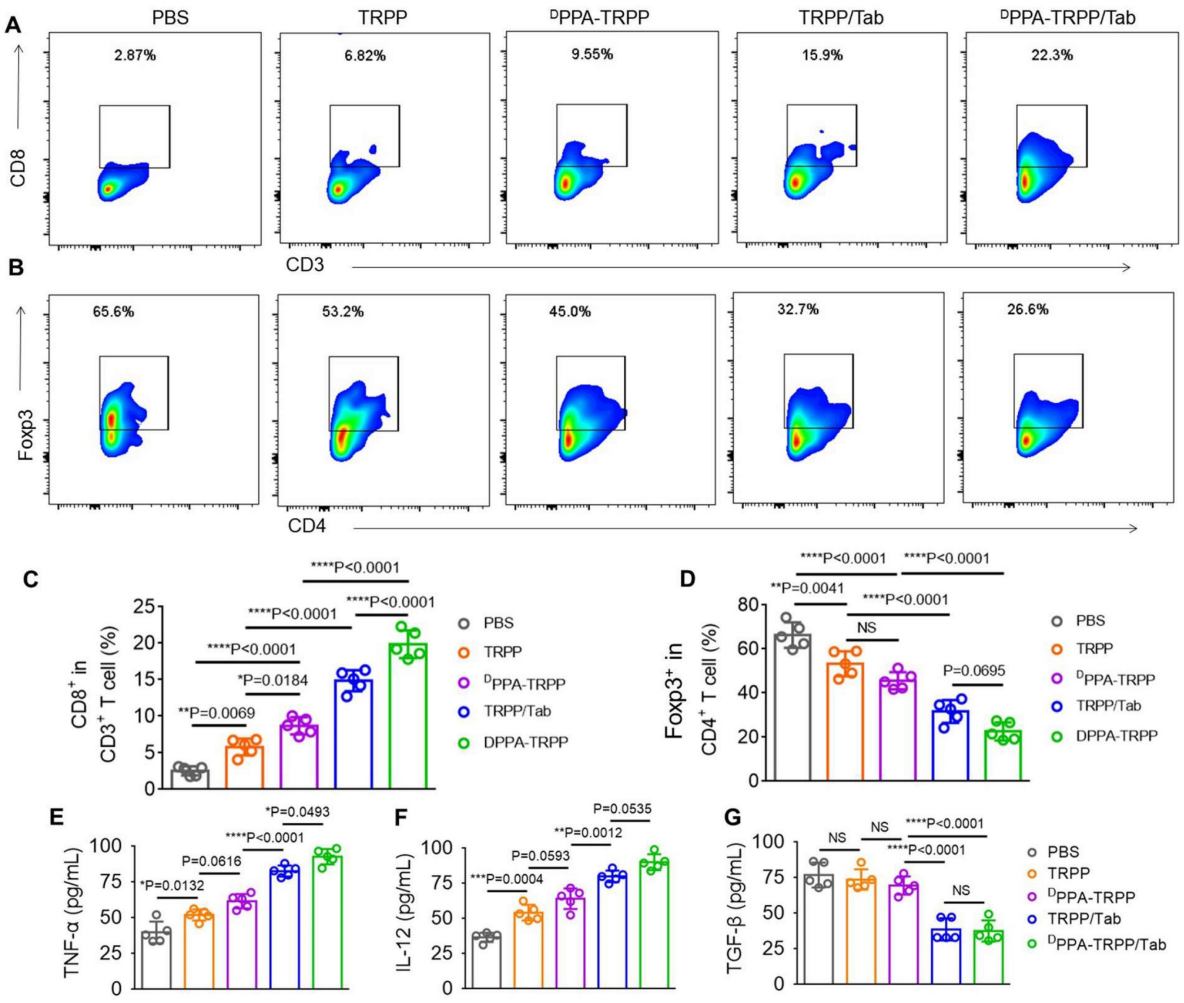
**Cell population analysis and cytokine detection at the therapeutic ending point. (A)** Representative CD8^+^ CD3^+^ T cell percentages and **(B)** CD4^+^ Foxp3^+^ percentages in tumor tissues. **(C)** Quantitative analyses of CD8^+^ CD3^+^ T cell and **(D)** CD4^+^ Foxp3^+^ percentages in tumor tissues after treatment measured by flow cytometry. Cytokines **(E)** TNF-α, **(F)** IL-12 and **(G)** TGF-β levels detection in tumor tissues. ^*^P < 0.05, ^**^P < 0.01, ^***^P < 0.001, ^****^P < 0.0001 by analysis of ANOVA with Tukey's post-hoc test.

To further study the antitumor activity, we investigated CAFs and T cell distribution, analyzed the T cell population in tumor tissue and detected cytokines at the therapeutic endpoint. As shown in Figure S29, significant CD8^+^ (red color) T cell infiltration was observed in tumor tissue of mice after ^D^PPA-TRPP/Tab treatment, while the α-SMA expression decreased, indicating effective inhibition of CAFs. Flow cytometry analyses were consistent with the above results that CD8^+^CD3^+^ T cell percentages notably increased (Figure 6A) and Foxp3^+^CD4^+^ T cell percentages significantly decreased (Figure 6B) in mice given the ^D^PPA-TRPP/Tab treatment. CD8^+^ T cell percentages in tumor tissue of mice treated with ^D^PPA-TRPP/Tab were 1.4-, 2.3-, 3.4- and 7.8-fold higher than those of TRPP/Tab, ^D^PPA-TRPP, TRPP and PBS treated mice, respectively (Figure 6C). Percentages of CD4^+^Foxp3^+^ T cells for mice treated with ^D^PPA-TRPP/Tab were 0.72-, 0.50-, 0.43- and 0.34-fold lower than those of TRPP/Tab, ^D^PPA-TRPP, TRPP and PBS treated mice, respectively (Figure 6D), demonstrating that Tab played a key role in facilitating Tregs' decrease. The highest TNF-α and IL-12 levels were detected in mice undergoing the ^D^PPA-TRPP/Tab treatment compared with mice in the other groups (Figure 6E, F). TGF-β levels were notably suppressed in mice treated with ^D^PPA-TRPP/Tab and TRPP/Tab, 0.51 to 0.56-fold lower than in mice treated with ^D^PPA-TRPP or TRPP alone, and 0.49 to 0.50-fold lower than in mice given PBS (Figure 6G). The results above indicate that multiple factors are contributing to the improved antitumor activity, including Tregs suppression, TGF-β down regulation, TNF-α and IL-12 increase, and CD8^+^ T cell infiltration.

## Conclusion

We developed a polymersome-micelle transformable pro-drug nanoplatform ^D^PPA-TRPP/Tab for TME modulation, ICD generation and immune checkpoint blockade. The property of transformability resolved the dilemma between whether to use hydrophilic drug release in the TME for immunosuppressive reversal, or a hydrophobic chemotherapeutic for intracellular release to cause ICD. Tab was released from TRPP in the TME, where TRPP re-assembled into prodrug micelles for further internalization by tumor cells to induce ICD. The conjugated DOX was the ICD inducer as well as the hydrophobic segment, the relatively low content of which ensured the biosafety of this nanoplatform. ^D^PPA-1 was able to shed from the TRPP surface when encountering a high concentration of H_2_O_2_ in the TME, and the act as an ICI. Compared with anti PD-L1, ^D^PPA-1 has a lower cost of production, which would endow it an advantage during clinical implementation. The ^D^PPA-TRPP/Tab nanoplatform improved the compound's tumor accumulation, suppressed CAFs formation, reduced immunosuppressive cytokine secretion, promoted CD8^+^ CD4^+^ T cell infiltration and decreased Tregs distribution, resulting in a potent antitumor effect. Remarkably, after ^D^PPA-TRPP/Tab treatment, there was a 60% CR ratio with prolonged survival time in 4T1 tumor-bearing mice when the initial tumor volume was around 100 mm^3^. The rechallenge-tumors were also notably suppressed compared with tumors in untreated mice. Even for mice with a larger initial tumor (~150 mm^3^), there was still a high antitumor activity observed after ^D^PPA-TRPP/Tab treatment as compared with mice in the other groups. Thus, this study provides a new type of prodrug nanoplatform with transformable morphology to simultaneously induce CAFs inhibition, ICD and immune checkpoint blockade. The results suggest a clinical translatability of this “all-in-one” transformable prodrug platform for the treatment of malignant tumors.

## Supplementary Material

Supplementary materials and methods, schemes, figures and table.
