# Supplementary material for: Transformable prodrug nanoplatform via tumor microenvironment modulation and immune checkpoint blockade potentiates immunogenic cell death mediated cancer immunotherapy
Source: Theranostics. 2023 Mar 21;13(6):1906–20. doi: 10.7150/thno.83912 (PMC10091884; doi:10.7150/thno.83912)
Supplement: Supplementary file 1 — Supplementary materials and methods, schemes, figures and table. [file thnov13p1906s1.pdf]

Supporting Information

Transformable prodrug nanoplatform *via* tumor microenvironment modulation and immune checkpoint blockade potentiates immunogenic cell death mediated cancer immunotherapy

Weijing Yang[1,2], Jinmeng Yi[3,4], Rongrong Zhu[1,2], Yichen Guo[1,2], Kaixin Zhang[1,2], Yongjian Cao[1,2], Xinyan Li[3,4], Jinjie Zhang[1,2], Zhenzhong Zhang[1,2], Yongjuan Li[3,4,*], Xiaoyuan Chen[5,6,7,*]

1. School of Pharmaceutical Sciences, Zhengzhou University, Zhengzhou 450001, China
2. Key Laboratory of Targeting Therapy and Diagnosis for Critical Diseases, Zhengzhou 450001, Henan Province, China
3. The center of Infection and Immunity, Academy of Medical Sciences, Zhengzhou University, Zhengzhou, Henan 450001, China
4. Medical Research Center, The First Affiliated Hospital of Zhengzhou University, Zhengzhou University, Zhengzhou, Henan 450001, China
5. Departments of Diagnostic Radiology, Chemical and Biomolecular Engineering, and Biomedical Engineering, Yong Loo Lin School of Medicine and Faculty of Engineering, National University of Singapore, Singapore, 117597 Singapore
6. Clinical Imaging Research Centre, Centre for Translational Medicine, Yong Loo Lin School of Medicine, National University of Singapore, Singapore 117599, Singapore
7. Nanomedicine Translational Research Program, NUS Center for Nanomedicine, Yong Loo Lin School of Medicine, National University of Singapore, Singapore 117597, Singapore

Materials

4-Cyano-4-(phenylcarbonothioylthio)pentanoic acid N-succinimidyl ester (CPPA-NHS) and 3, 3'-dithiodipropionic acid (DTPA, 99%) was purchased from TCI (Shanghai) Development Co. Ltd. 2-(Hexamethyleneimino) ethanol (C7A) was purchased from Alfa Aesar Company. Methacryloyl chloride (97%), (2-boc-amino)ethyl methacrylate (BAEMA, 99%), sodium borohydride (96%), selenium powder (99%), 2-bromoethanol (95%), azobisisobutyrontrile (AIBN, 98%), N-(3-dimethylaminopropyl)-N-ethylcarbodiimide hydrochloride (EDC·HCl, 98%), dimethylaminopyridine (DMAP, 99%), N-hydroxysuccinimide (NHS, 98%), thiazolyl blue tetrazolium bromide (MTT, 97.5%) were purchased from Sigma-Aldrich. Acid-poly(ethylene glycol)-amine (COOH-PEG-NH_2, M_w = 5.0 kg/mol) was purchased from Biochempeg. D-peptide antagonist (^{D}PPA-1, 98%) was purchased from Shanghai TOP-Peptide Biotechnology Co. Ltd. Glutathione (GSH) was purchased from VWR Corporation. Talabostat mesylate (Tab, 99%) and Doxorubicin hydrochloride (DOX, 99%) were purchased from MedChemExpress. Oxi Vision Green™ Hydrogen Peroxide Sensor was purchased from AAT Bioquest (Sunnyvale, CA). 1,1-dioctadecyl-3,3,3,3-tetramethylindotricarbocyanine (DIR) was purchased from BIOMART. CN. 4', 6-Diamidino-2-phenylindole (DAPI), collagenase IV and DNAse, 2, 4, 6-trinitrobenzene sulfonic acid (TNBSA) was purchased from Thermo Fisher Scientific. Anti CD31 and anti CD3 were purchased from Santa Cruz. Anti-calreticulin (CRT), anti CD8 and anti CD4 were purchased from Bioss Antibodies. Goat anti-Rat IgG (H+L) Secondary Antibody Alexa Fluor™ 488, and Donkey anti-Rabbit IgG (H+L) Secondary Antibody Alexa Fluor™ 594 were purchased from Thermal Fisher Scientific. Alexa Fluor® 488 anti-CRT, Alexa Fluor® 647 anti-HMGB1 and α-SMA (primary antibody) were purchased from Abcam. PE anti CD11c, PerCP Cy5.5 anti CD80, APC anti CD86, APC anti CD8a, PE anti CD3e, Percp Cy5.5 anti CD4, FITC anti Foxp3, FITC anti CD44, were purchased from BioLegend. Tissue-Tek O.C.T. Compound and 4% Paraformaldehyde Fix Solution were purchased from Solarbio Life Sciences. HMGB1 ELISA kit, TGF-β, IL-12, TNF-α were purchased from R&D system.

Characterization

The ^{1}H NMR spectra were carried out on a Bruker Avance 400 spectrometer. Dynamic light

scattering (DLS) measurements were performed on Nano ZS Zetasizer (DLS, Zetasizer Nano ZEN3600, Malvern). Transmission electron microscopy (TEM) was executed with Hitachi HT7700 transmission electron microscope. The UV-vis absorbance and fluorescence emission spectra detection were performed on a UV-2600 spectrophotometer (SHIMADZU) and RF-5301PC fluorometer (SHIMADZU), respectively. The Methyl Thiazolyl Tetrazolium (MTT) assays were executed in microplate reader (Biotek Synergy H1). Cells images were acquired by confocal laser scanning microscope (CLSM) (Leica TCS SP8 STED). Flow cytometry analysis was executed on BD Beckman Coulter flow cytometer (Brea, CA) and FlowJ software. *In vivo* tumor accumulation was monitored by IVIS spectrum (IVIS Lumina XRMS Series III).

Synthesis of monomer MA-C7A

2-(Hexamethyleneimino) ethanol (14.3 g, 0.1 mol) and TEA (10.1 g, 0.1 mol) was dissolved in tetrahydrofuran (THF, 50 mL), and then methacryloyl chloride (10.4 g, 0.1 mol) was added dropwise. The mixture was stirred at 70 ºC for 6 h and then filtered to remove the precipitation. The final product (MA-C7A) was obtained as colorless liquid by *via* distillation in vacuo (83~87 ºC) (13.7 g, yield 65%). The ^{1}H NMR spectrum was shown in Figure S1.

Synthesis and characterization of 2, 2'-diselanediylbis(ethan-1-ol) (HO-Se-Se-OH, dSe)

Sodium borohydride (3.8 g, 100 mmol) was dissolved in water (30 mL), into which selenium powder (6.4 g, 81 mmol) was added under nitrogen atmosphere at room temperature (RT). The mixture was stirred for 30 min at 105 °C. An additional equivalent of selenium powder (6.4 g, 81 mmol) was added, and the mixture was stirred for another 30 min at 105 °C. After the mixture was cooled to RT, 2-bromoethanol (12.5 g, 100 mmol) dissolved in THF (50 mL) was added under nitrogen atmosphere and stirred for another 24 h. The final product was purified by column chromatography on silica gel and obtained as yellow liquid (6.2 g, yield 21.3%) (Figure S5, Figure S9, Supporting Information). The structure changes of dSe with or without H_2O_2 treatment were characterized by Fourier-transform infrared spectroscopy (FT-IR) (Figure S10, Supporting Information).

***In vitro* drug loading and release behavior study**

Fibroblast activation protein (FAP) inhibitor talabostat loaded nanomedicine TRPP/Tab and ^{D}PPA-TRPP/Tab was also prepared by solvent-exchange methodology, when Tab in DMSO (10 μL, 10 mg/mL) pre-mixed with dSe-PEG-PC7A-P(BAEMA-(AEMA-SS-DOX)) in THF (200 μL, 5 mg/mL) needed before adding into PBS. After volatilization and dialysis to remove THF and unloaded drug, TRPP/Tab was successful fabrication, size of which was measured by DLS. Drug loading content (DLC) and drug loading efficiency (DLE) were measured by liquid chromatography-mass spectrometry (LC-MS) (WatersUplc-XEVQTQD) and calculated *via* the following formula:

DLC (wt.%) = (weight of loaded drug/total weight of loaded drug and polymer) × 100%

DLE (%) = (weight of loaded drug/weight of drug in feed) × 100%

In vitro DOX release was studied when ^{D}PPA-TRPP was put in the release bags (MWCO, 12000) placing in a shaking bed (37 °C, 200 rpm). To obtain the sink condition, 0.5 mL TRPP in each bag was surrounded by 25 mL release media. At pre-set time points, 5 mL release media was taken out with the same volume fresh replacement. The following used buffer concentration was 10 mM containing with 150 mM NaCl if no specific illustration. The media were PBS (pH 6.8, 10 mM GSH), PBS (pH 6.8, no GSH), PBS (pH 7.4, 10 mM GSH), PBS (pH 7.4, no GSH), acetic acid/sodium acetate buffer (HAc/NaOAc, pH 5.0, 10 mM GSH), HAc/NaOAc (pH 5.0, no GSH), respectively. The released DOX was measured by fluorescence photometer.

In vitro Tab release behavior was explored in a similar method with that of DOX, only difference in release condition. The media were PBS (pH 6.8, no H_2O_2), PBS (pH 6.8, 100 μM H_2O_2), PBS (pH 7.4, no H_2O_2), PBS (pH 7.4, 100 μM H_2O_2), respectively. The released Tab was detected by LC-MS.

Cytotoxicity and cellular internalization of ^{D}PPA-TRPP in 4T1 cells

TRPP cytotoxicity in 4T1 cells was investigated by MTT assays. Shortly, 4T1 cells (5.0×10^{3}/well) were seeded in 96-well plates. After growth for 24 h, free DOX and TRPP (TRPP was pretreated with pH 6.8 and 100 μM H_2O_2, which was the condition if no specific mention in

the following cell experiments.) were separately added, when drug concentrations from low to high were 0.16, 0.32, 0.62, 1.25, 2.5, 5, 10 μg/mL. After incubation for 48 h, MTT solution (10 μL, 5 mg/mL) was added. After 4 h incubation, the media were aspirated from each well with 150 μL DMSO addition. Absorbance at 570 nm was detected by microplate reader.

The cellular internalization was characterized by CLSM and flow cytometry, respectively. As to CLSM characterization, 4T1 cells (2.0×10^4/well) were seeded in 24-well plate. After 24 h growth, ^{D}PPA-TRPP, TRPP and free DOX (drug Conc.: 5 μg/mL) were added incubation for 2, 4, and 8 h, respectively. Cells were firstly washed by PBS followed with 4% paraformaldehyde fixation for 15 min. After PBS washing again, cell nuclei were stained with DAPI (2 μg/mL) for 10 min. After further PBS washing, cells were covered by coverslip and sealed with nail polish. Cell images were captured by CLSM. As for flow cytometry characterization, 4T1 cells (5.0×10^5/well) were seeded in 6-well plate growth for 24 h. ^{D}PPA-TRPP, TRPP and free DOX were separately added incubation for 4 h and 8 h. Cells went through trypsinization, PBS washing, centrifugation. Eventually, PBS suspension (0.5 mL/sample) was tested by flow cytometer.

Immunogenic cell death (ICD) mediated by ^{D}PPA-TRPP in 4T1 cells

^{D}PPA-TRPP mediated ICD were detected by calreticulin (CRT) exposure and High Mobility Group Box 1 (HMGB1) release. For CRT exposure detection, CLSM and flow cytometry were separately characterized. In short, 4T1 cells were seeded in 24-well plate. After 24 h culture, ^{D}PPA-TRPP, TRPP, TP and free DOX were separately added for 24 h incubation. Cells were washed by PBS, fixed by 4% paraformaldehyde, stained with Alexa Fluor® 488 anti-CRT. After PBS washing, cell nuclei were stained with DAPI. Cell images were acquired by CLSM. For flow cytometry characterization, 4T1 cells (5.0×10^5/well) were seeded in 6-well plate culture for 24 h for flow cytometry characterization. After ^{D}PPA-TRPP, TRPP and free DOX addition incubation for 24 h, cells underwent trypsin digestion, centrifugation, Alexa Fluor® 488 anti-CRT staining for 40 min at r.t. After further PBS washing and centrifugation, cells in each group suspended in 0.5 mL PBS with flow cytometer measurement.

As to HMGB1 release investigation, CLSM and ELISA were separately exploited. For CLSM characterization, 4T1 cells were firstly seeded in 24-well plate (2.0×10^4/well) growing for 24 h.

Subsequently, ^{D}PPA-TRPP, TRPP, TP and free DOX were added, respectively. After incubation for 48 h, cells underwent PBS washing, 4% paraformaldehyde fixation, Alexa Fluor® 647 anti-HMGB1 staining and cell nuclei staining. Cell images were obtained by CLSM. For ELISA measurement, 4T1 cells (1.0×10^4/well) were seeded in 96-well plate culture for overnight. ^{D}PPA-TRPP, TRPP, TP and free DOX were added, respectively (n = 4). After 48 h incubation, supernatant in each well was aspirated when released HMGB1 from treated cells was detected by ELISA according to manufacturer's procedure.

In vitro immunity of TP, TRPP and ^{D}PPA-TRPP for DC maturation investigation

As for immunity of nanocarrier itself without DOX conjugation, TP and PBS were used to investigate DC maturation by flow cytometer. Shortly, 4T1 cells were seeded in 6-well plate culturing overnight. TP and PBS were then separately added into each well incubation for 24 h. After digestion, centrifugation and staining by PE anti CD11c, Percp Cy5.5 anti CD80 and APC anti CD86 for 40 min, cells were finally suspended in PBS (0.5 mL/sample) and tested by flow cytometer.

^{D}PPA-TRPP andTRPP induced ICD with DAMPs release which also was able to lead to DC maturation. In short, 4T1 cells were seeded in 6-well plate. After culturing overnight, ^{D}PPA-TRPP, TRPP and PBS were added, respectively. After 24 h incubation, the treated 4T1 cells were co-incubated with DC 2.4 cells for another 24 h. Subsequently, cells suffered from digestion, centrifugation, staining, suspension and detection by flow cytometer.

In vivo tumor accumulation investigation

For the convenient *in vivo* monitoring, Tab was replaced with fluorescence dye DIR encapsulation in nanoformulation during the *in vivo* tumor accumulation study. BALB/c mice were inoculated with 4T1 cells at the right flank. When tumor volume increased to 150~200 mm^3, mice were separately administered with ^{D}PPA-TRPP/DIR or TRPP/DIR. Tumor accumulation was monitored by IVIS spectrum at 2, 4, 8, 24 h post-injection.

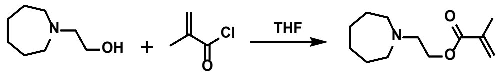

Scheme S1. Synthetic route of seven-ring monomer (MA-C7A).

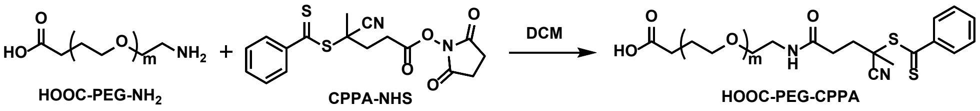

Scheme S2. Synthetic route of COOH-PEG-CPPA.

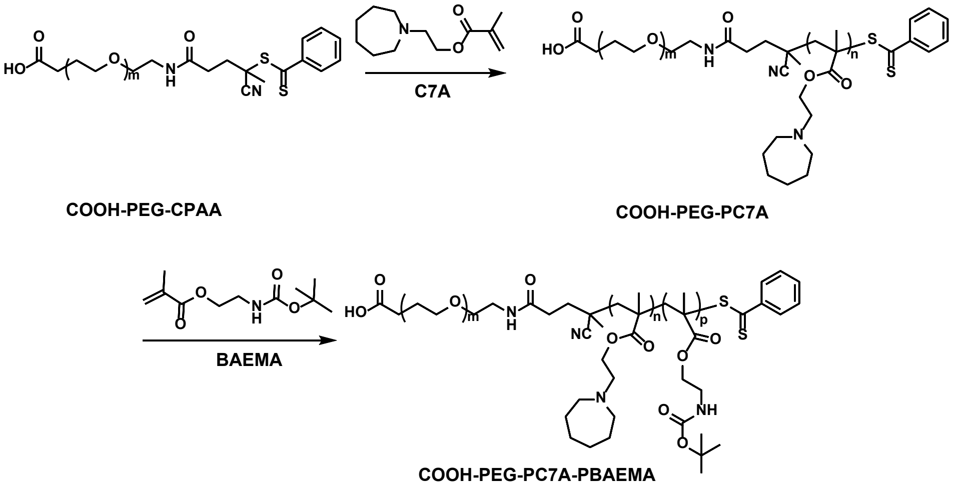

Scheme S3. Synthetic route of COOH-PEG-PC7A and COOH-PEG-PC7A-PBAEMA.

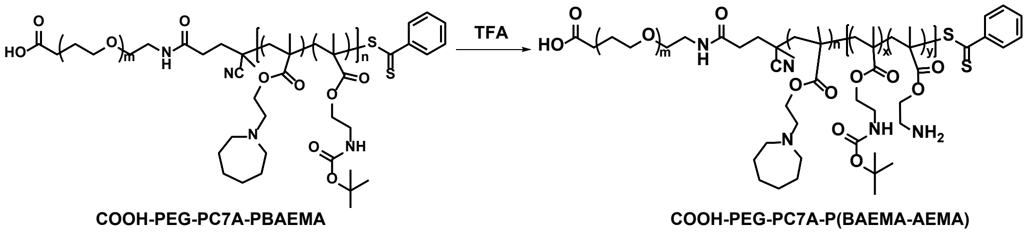

Scheme S4. Synthetic route of COOH-PEG-PC7A-P(BAEMA-AEMA).

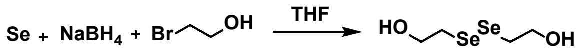

Scheme S5. Synthetic route of 2,2'-diselanediylbis(ethan-1-ol) (Se-Se, dSe).

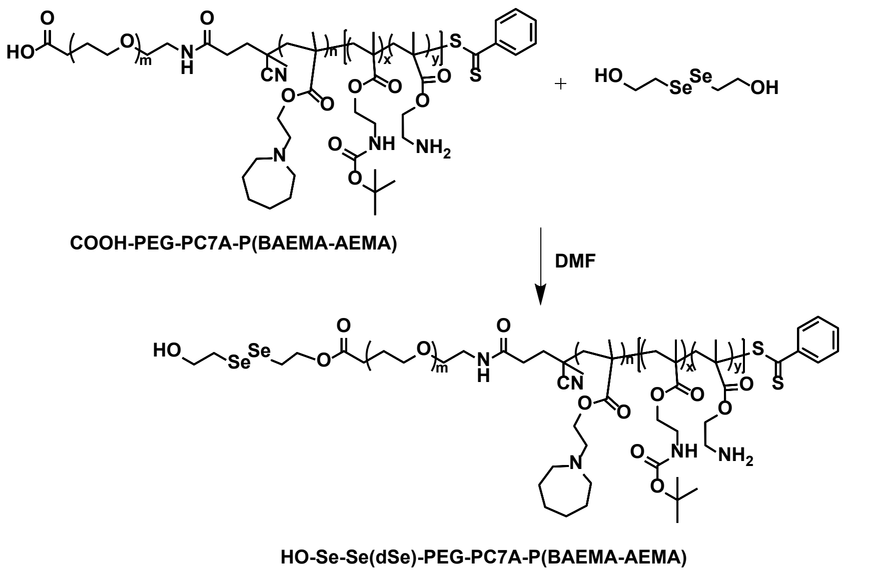

Scheme S6. Synthetic route of HO-Se-Se(dSe)-PEG-PC7A-P(BAEMA-AEMA).

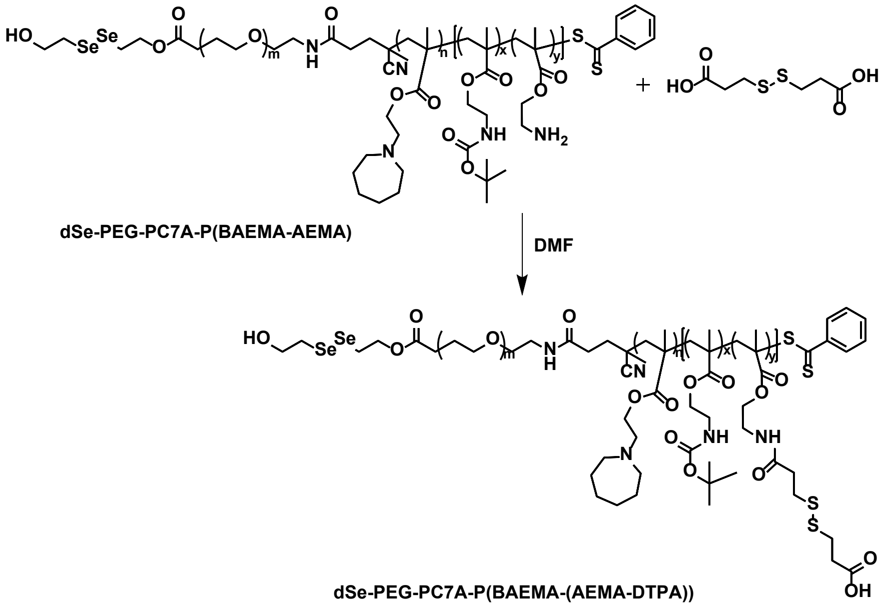

Scheme S7. Synthetic route of dSe-PEG-PC7A-P(BAEMA-(AEMA-DTPA)).

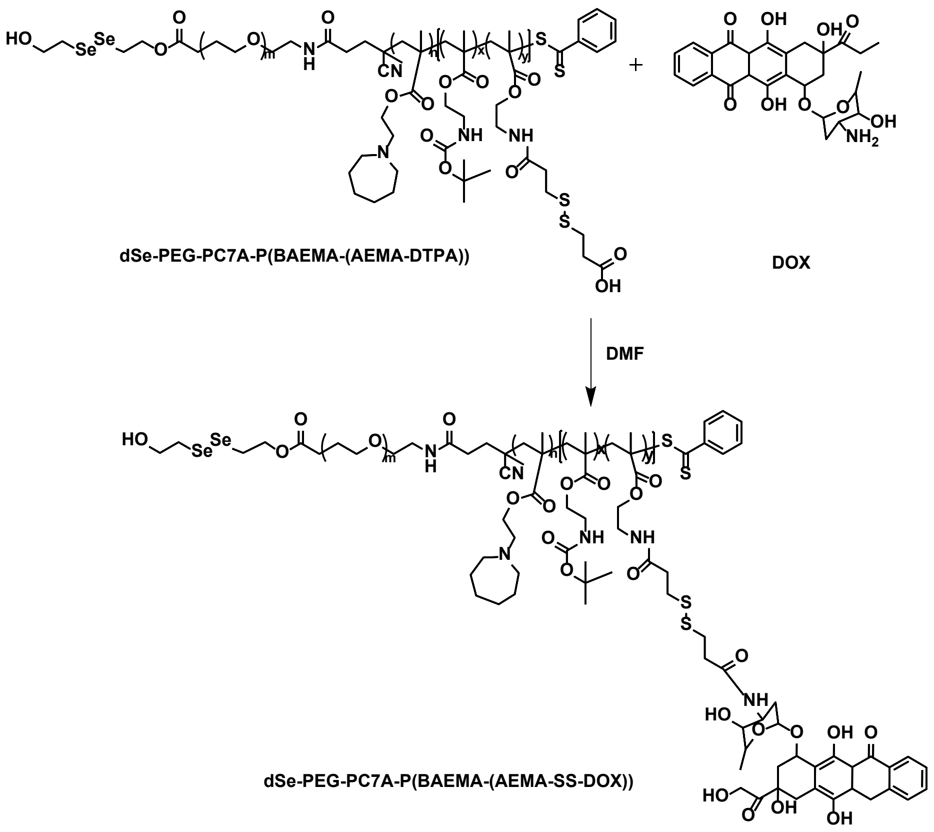

Scheme S8. Synthetic route of dSe-PEG-PC7A-P(BAEMA-(AEMA-DOX)).

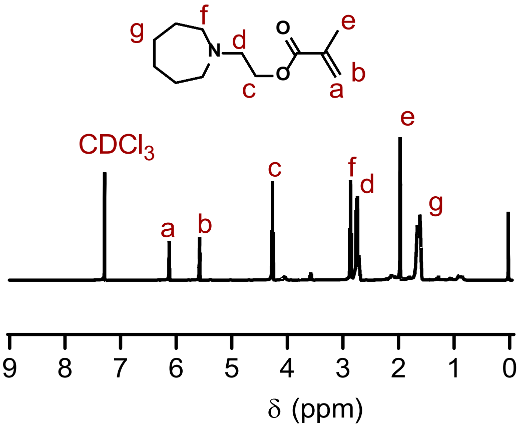

Figure S1. ^{1}H NMR spectrum (400 MHz, $CDCl_3$) of monomer MA-C7A.

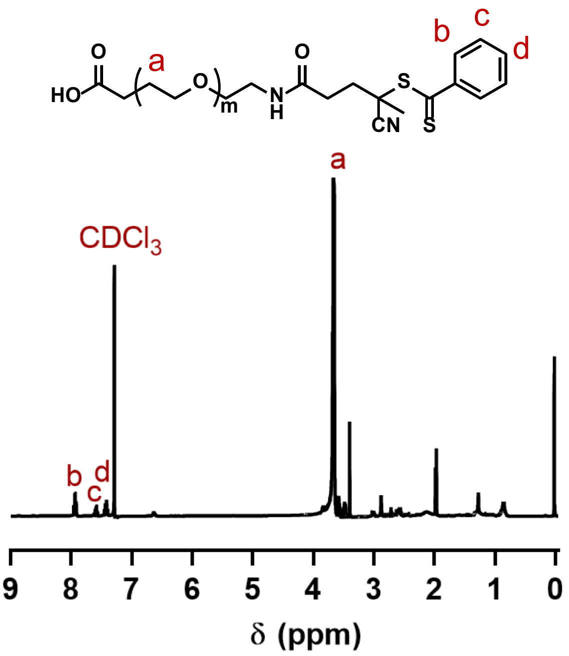

Figure S2. ^{1}H NMR spectrum (400 MHz, $CDCl_3$) of monomer PEG-CPPA.

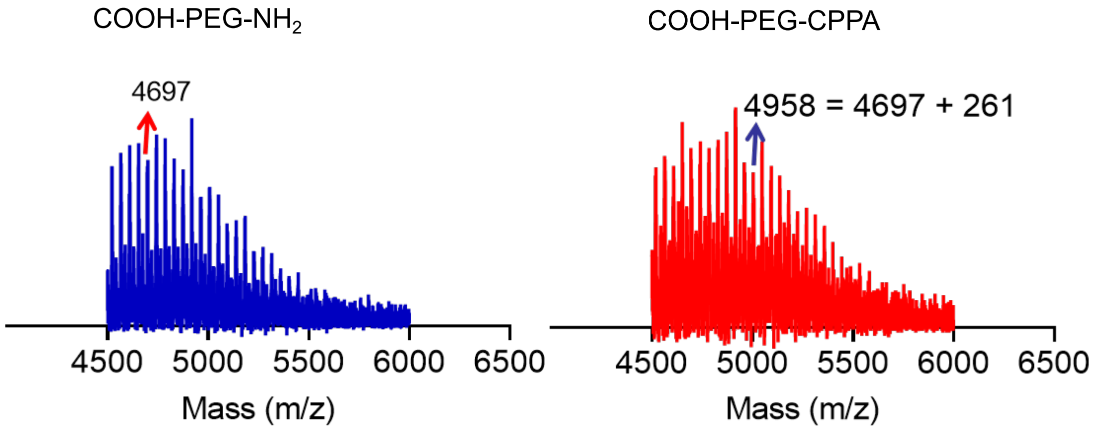

Figure S3. MALDI-TOF mass spectrum of PEG-CPPA.

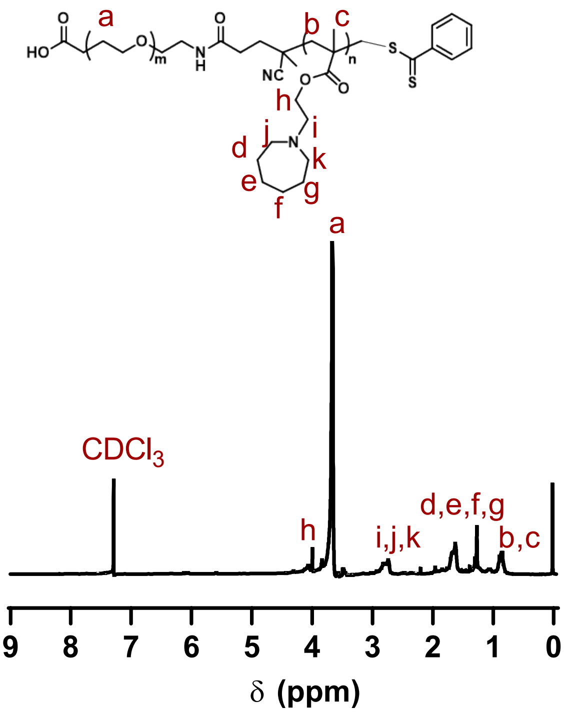

Figure S4. ^{1}H NMR spectrum (400 MHz, $CDCl_3$) of COOH-PEG-PC7A.

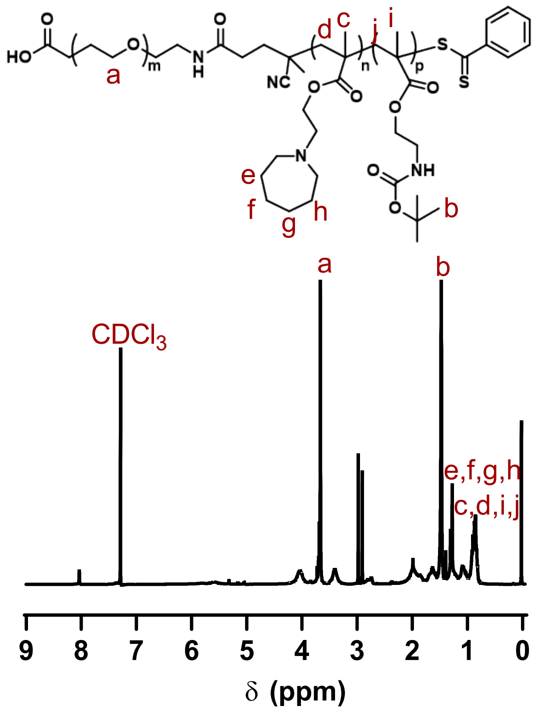

Figure S5. ^{1}H NMR spectrum (400 MHz, $CDCl_3$) of COOH-PEG-PC7A-PBAEMA.

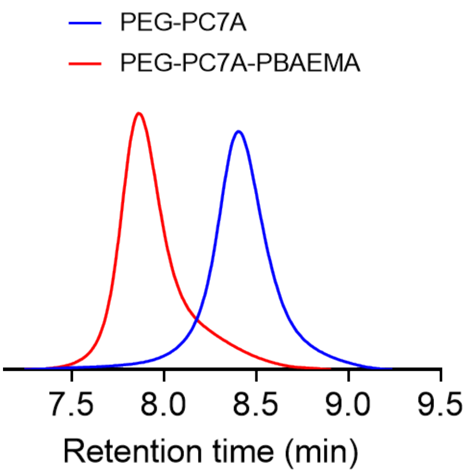

Figure S6. GPC traces of PEG-PC7A and PEG-PC7A-PBAEMA.

Table S1. Characteristics of block copolymers.

Entry	Copolymers	M_n (kg/mol)		GPC [b]	
		Design	^{1}HNMR[a]	M_n (kg/mol)	M_w/M_n [b]
1	PEG-PC7A	5.0-3.0	5.0-2.3	9.3	1.22
2	PEG-PC7A-PBAEMA	5.0-2.3-6.0	5.0-2.3-4.7	11.8	1.24

[a] Calculated from ^{1}H NMR (400 MH$_Z$, $CDCl_3$).

[b] Esterified with trifluoroacetic anhydride (TFAA) and then analyzed by GPC measurements using DMF as an eluent at a flow rate of 1.0 mL/min (standards: polystyrene, 30 °C).

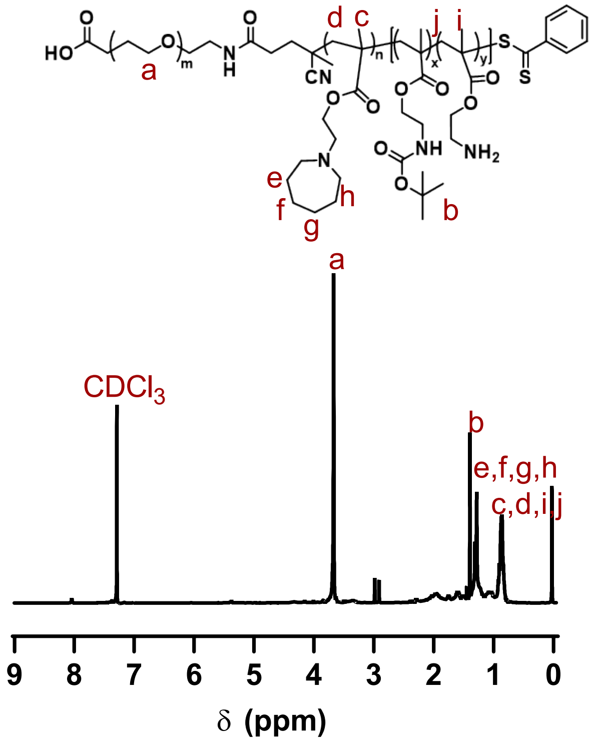

Figure S7. ^{1}H NMR spectrum (400 MHz, $CDCl_3$) of COOH-PEG-PC7A-P(BAEMA-AEMA).

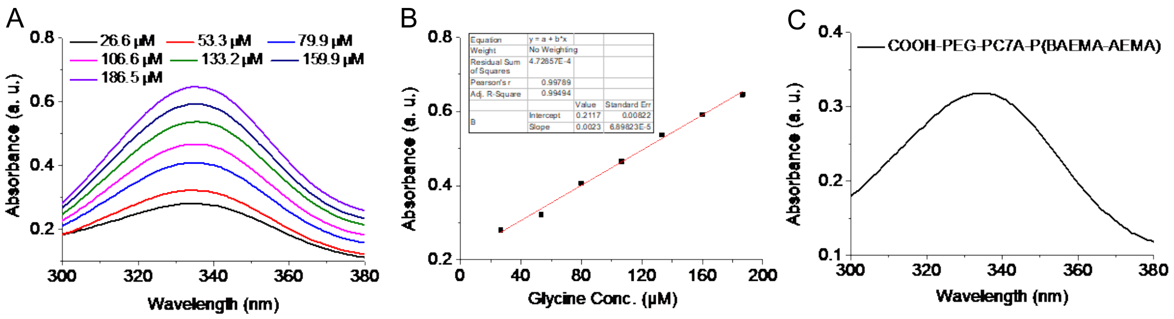

Figure S8. TNBSA method to detect the primary amine content in the hydrolyzed copolymer.

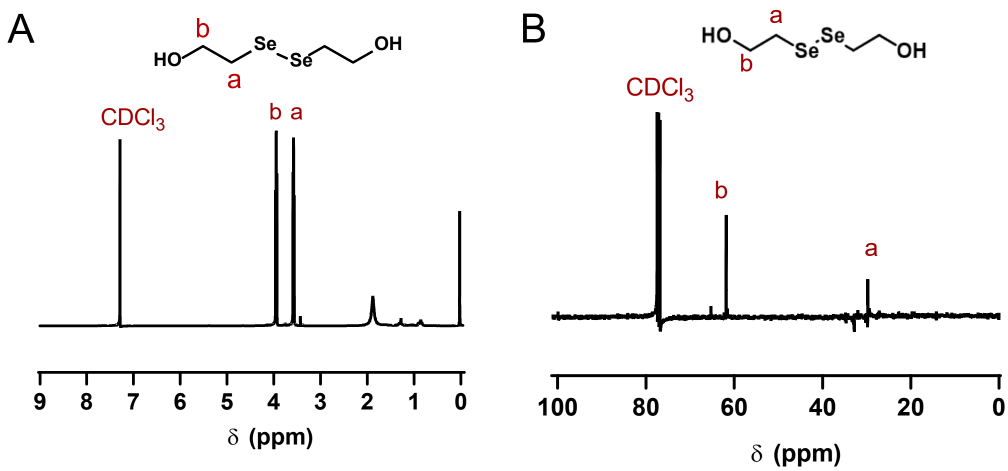

Figure S9. ^{1}H NMR and ^{13}C NMR spectra (400 MHz, $CDCl_3$) of dSe.

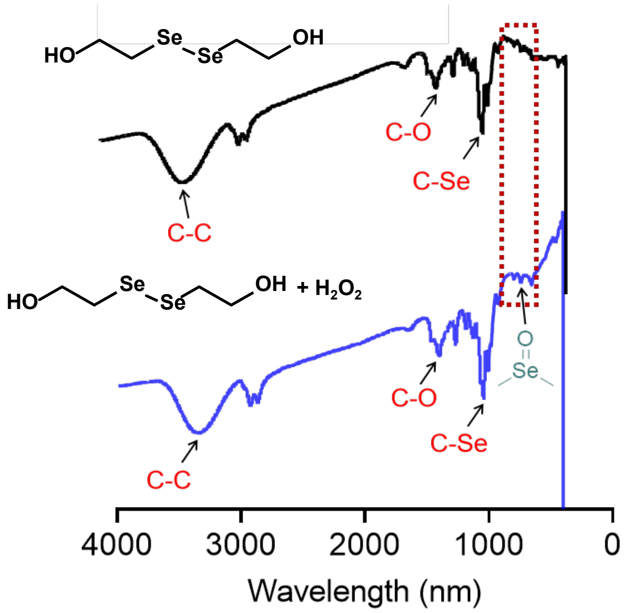

Figure S10. Infrared (IR) spectra of dSe in the absence and existence of H_2O_2 (100 μM).

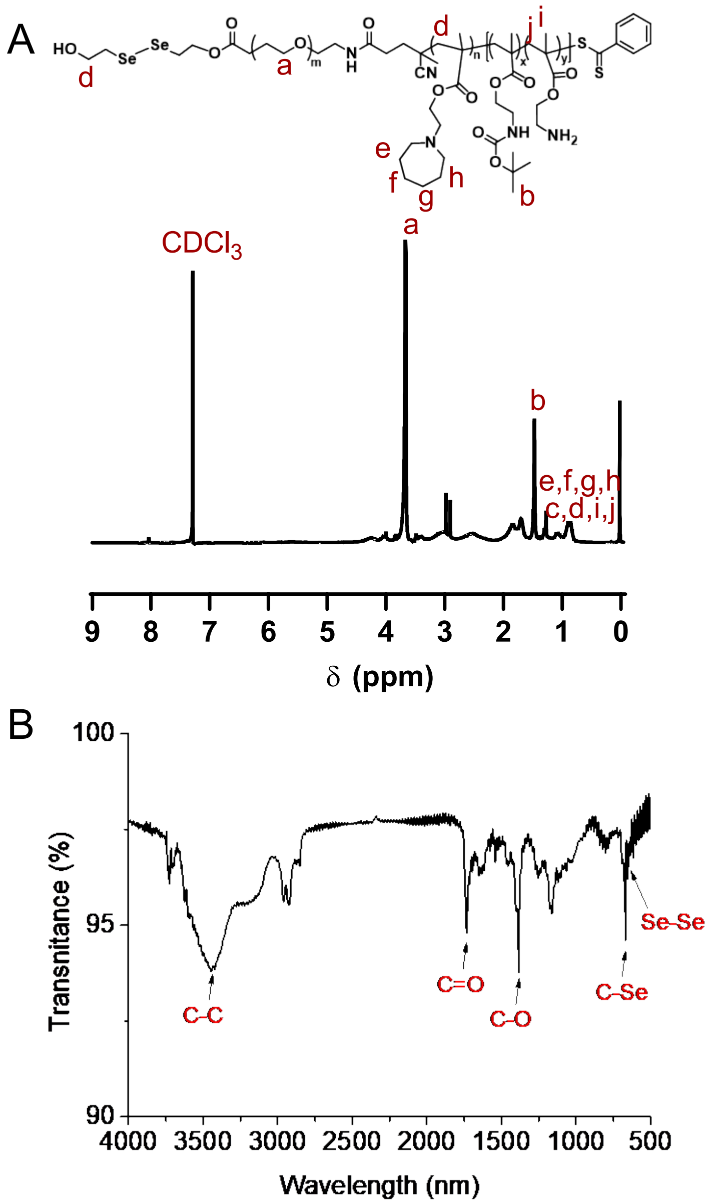

Figure S11. ^{1}H NMR (400 MHz, $CDCl_3$) and IR spectra of dSe-PEG-PC7A-P(BAEMA-AEMA).

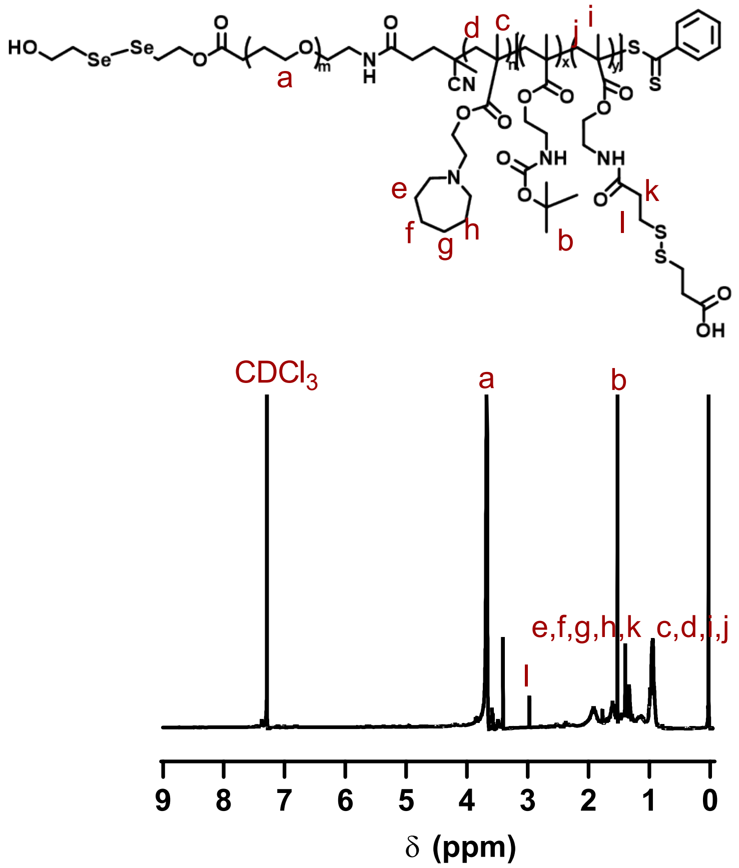

Figure S12. ^{1}H NMR spectrum (400 MHz, $CDCl_3$) of dSe-PEG-PC7A-P(BAEMA- (AEMA-DTPA).

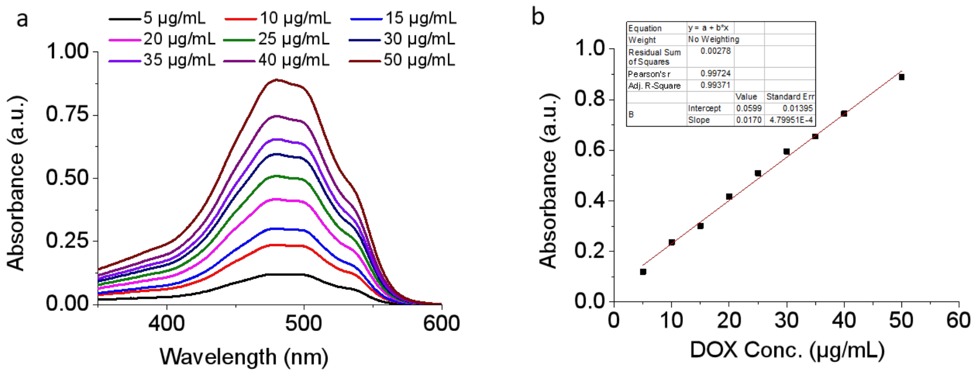

Figure S13. Free DOX absorbance spectra (a) standard curve (b) measured by UV-vis.

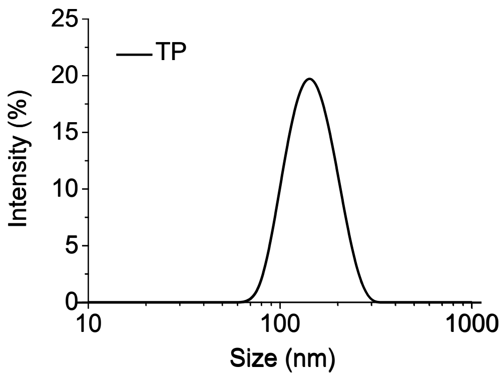

Figure S14. Size and size distribution of TP measured by dynamic light scattering (DLS).

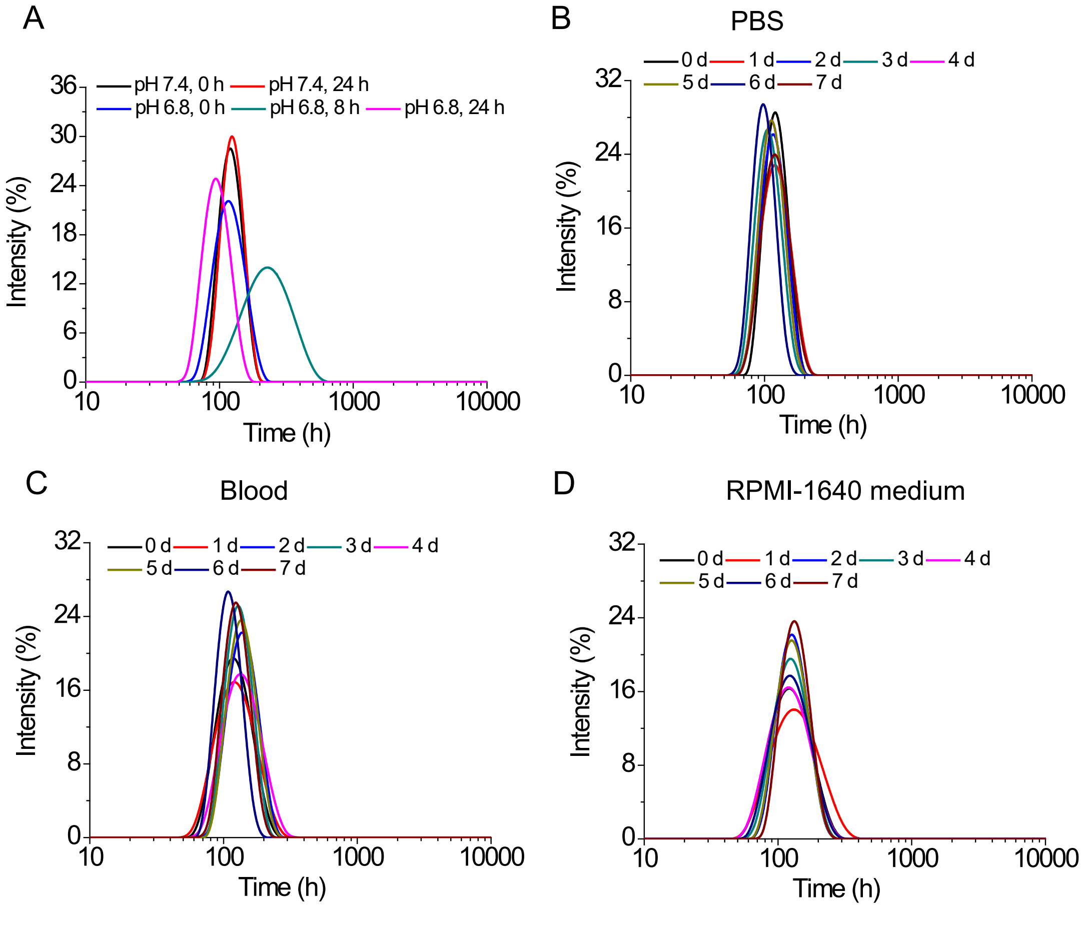

Figure S15. pH responsiveness and stability investigation of ^{D}PPA-TRPP monitored by DLS.

Table S2. Characteristics of TRPP/Tab and ^{D}PPA-TRPP/Tab

Polymersomes	Theor. DLC (wt.%)	Size [a] (nm)	PDI [a]	DLC [b] (wt.%)	DLE [b] (%)
TRPP/Tab	5	118 ± 3	0.18 ± 0.04	4.10	81.3
	10	120 ± 6	0.21 ± 0.03	6.65	64.1
	15	132 ± 4	0.22 ± 0.05	8.27	51.1
^{D}PPA-TRPP/Tab	5	121 ± 4	0.19 ± 0.02	4.27	84.8
	10	125 ± 4	0.22 ± 0.04	6.62	63.8
	15	128 ± 8	0.21 ± 0.06	8.57	53.1

[a] Determined using Zetasizer Nano-ZS (Malvern Instruments) at 25 oC in PB (10 mM, pH 7.4).

[b] Determined by LC-MS measurement.

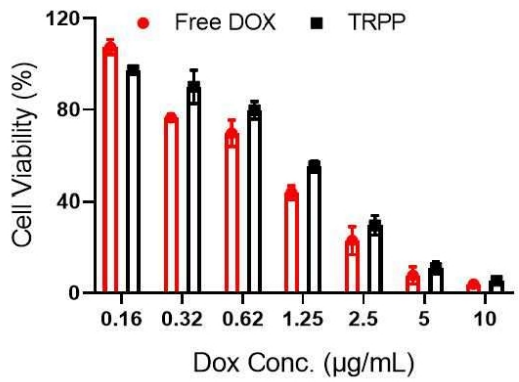

Figure S16. Cytotoxicity investigation of TRPP in 4T1 cells *via* MTT assays (n = 4).

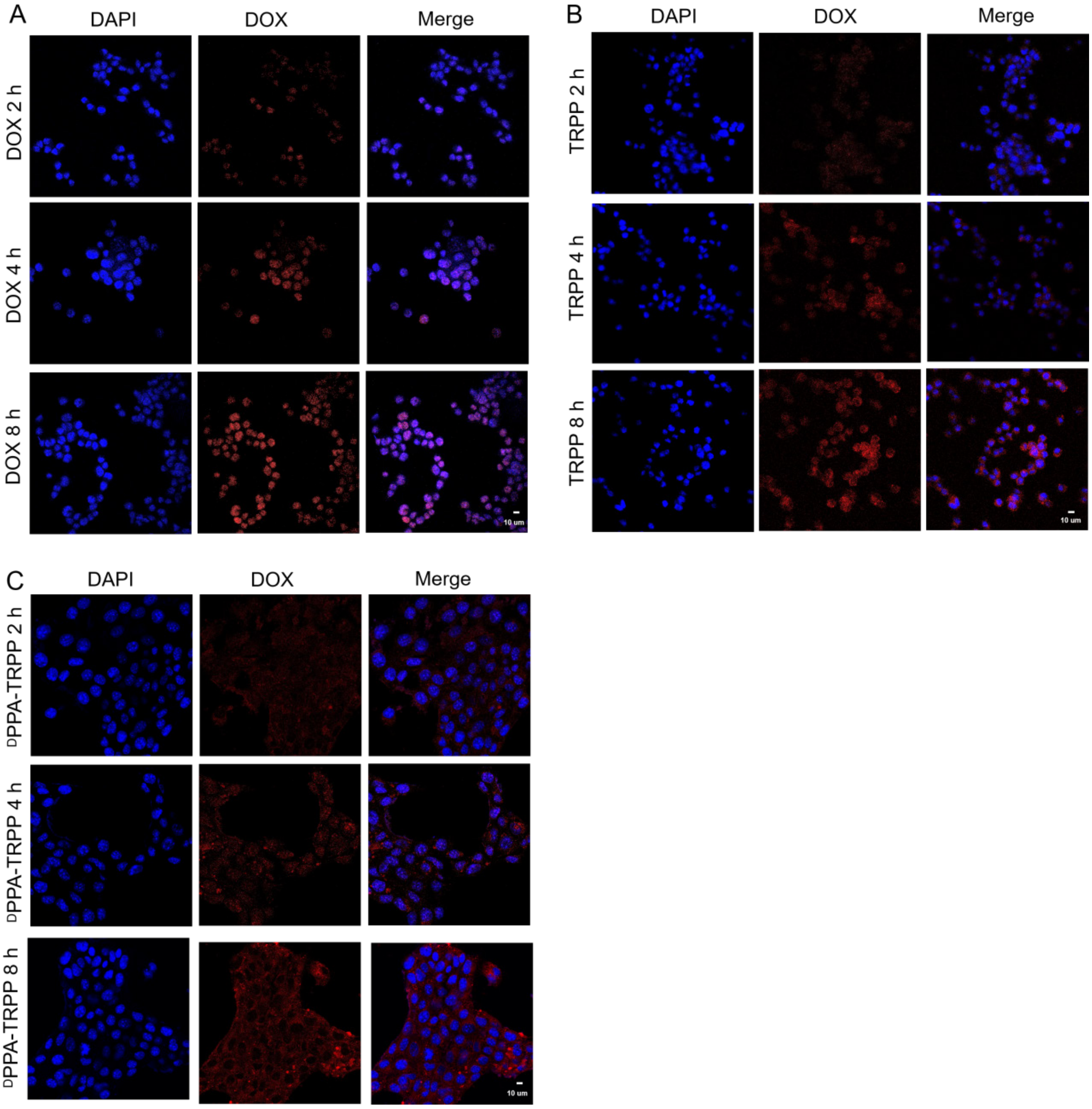

Figure S17. Cellular internalization of free DOX (A), TRPP (B) and ^{D}PPA-TRPP (C) in 4T1 cells at different time points *via* CLSM characterization.

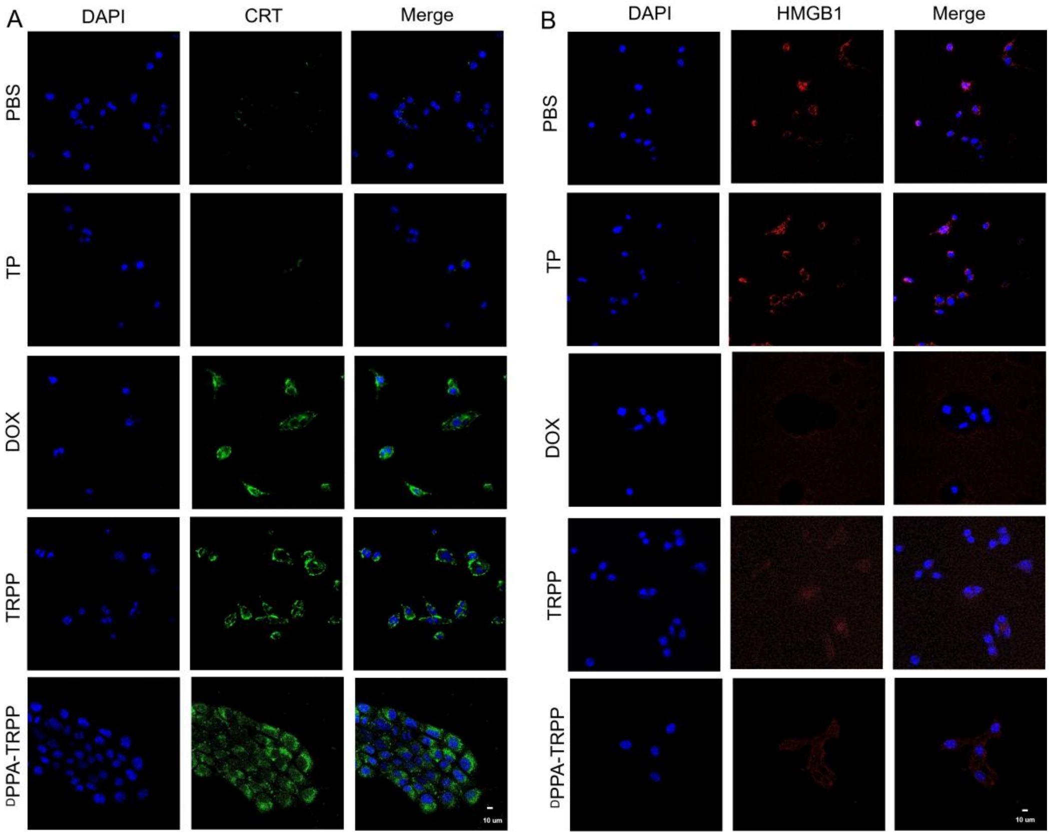

Figure S18. DPPA-TRPP induced ICD investigation in 4T1 cells characterized by CLSM. (A) CRT exposure characterization. (B) HMGB1 release behavior.

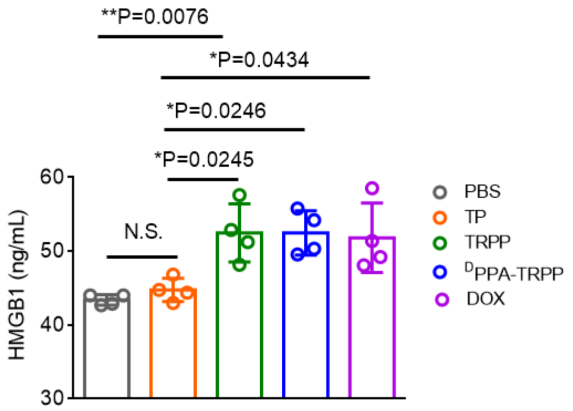

Figure S19. DPPA-TRPP induced HMGB1 release in 4T1 cells *via* ELISA for ICD investigation (n = 4). $*P < 0.05$, $**P < 0.01$ by analysis of ANOVA with Turkey's post-hoc test.

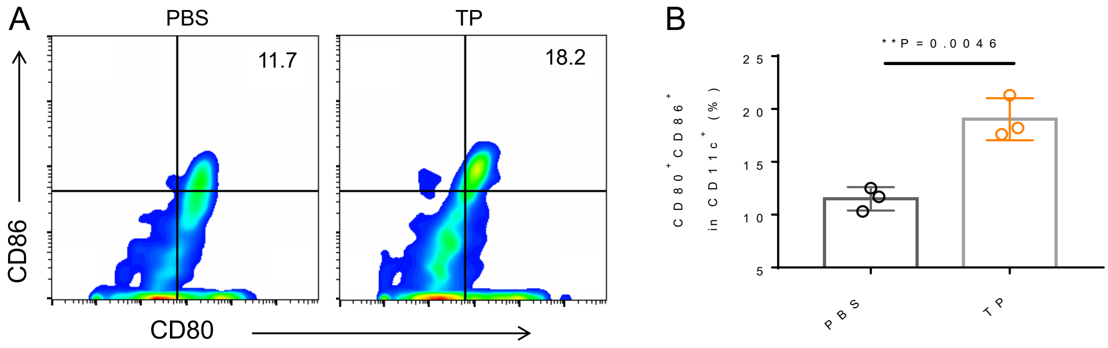

Figure S20. TP mediated DC maturation investigation. (A) Representative flow cytometry data. (B) Statistical data. Data presented as mean ± s.d. (n = 3). Statistical significance between the indicated groups was determined using two-sided unpaired t-tests. **P < 0.01

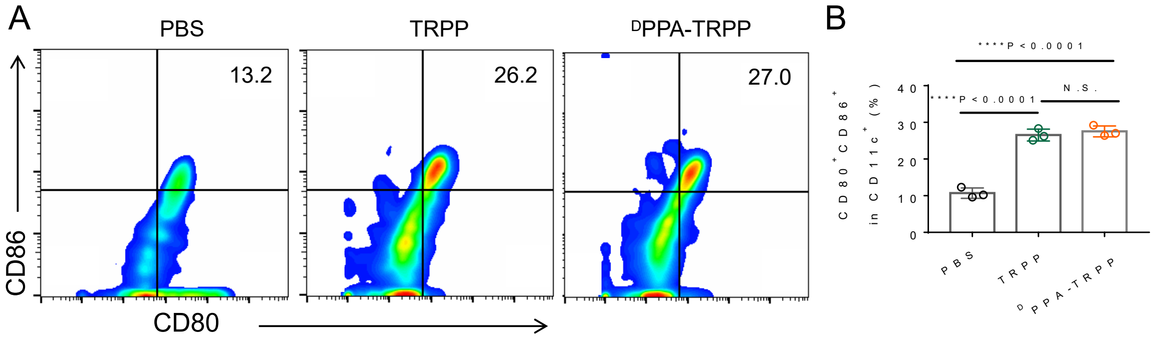

Figure S21. ICD-associated DAMPs of TRPP and ^{D}PPA-TRPP from 4T1 cells induced DC maturation. (A) Representative flow cytometry data. (B) Statistical data. Data presented as mean ± s.d. (n = 3). ****P < 0.0001 by analysis of ANOVA with Turkey's post-hoc test.

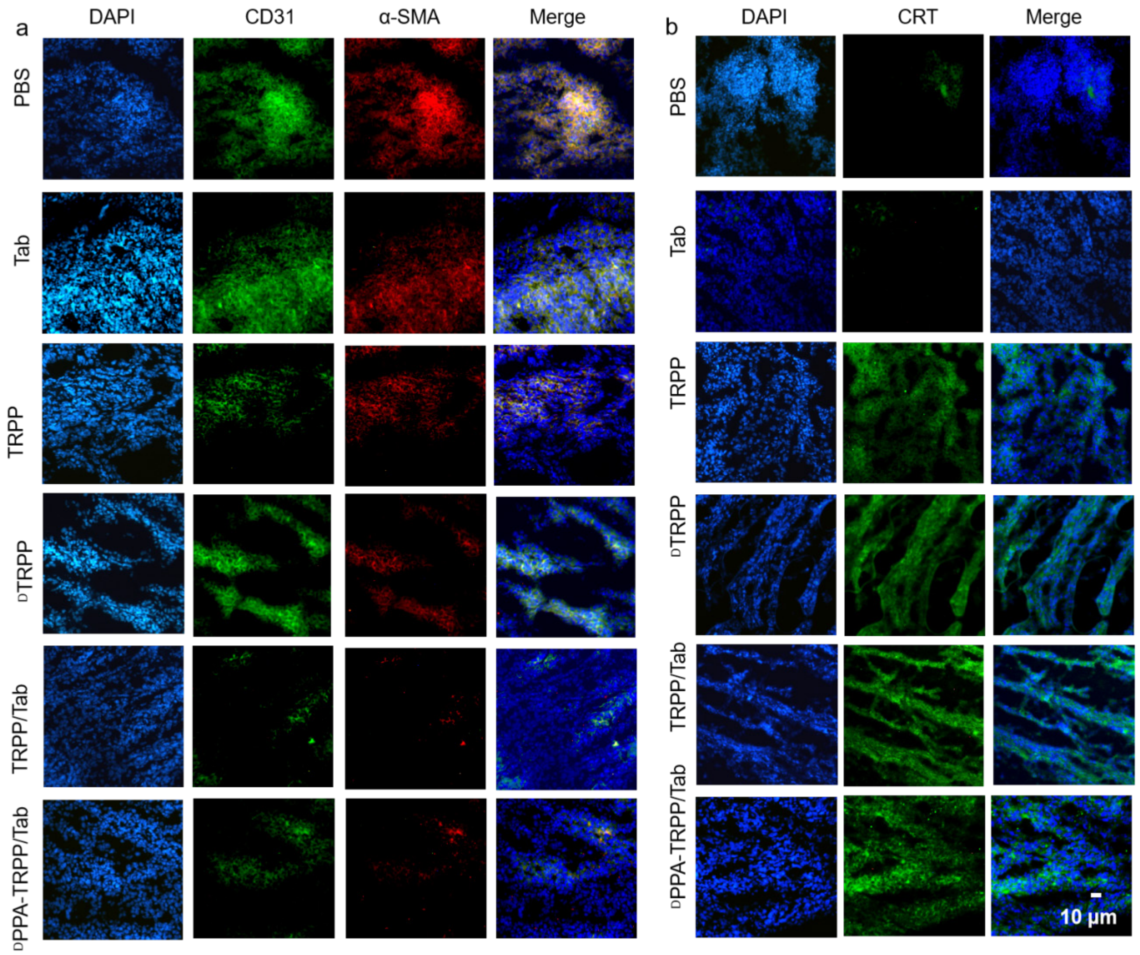

Figure S22. TME modulation and *in vivo* ICD characterization for 4T1 tumor bearing mice at day-6 post treatment. (a) α-SMA expression changes (red colors). Green represents blood vessels. (b) CRT exposure, green color presents CRT.

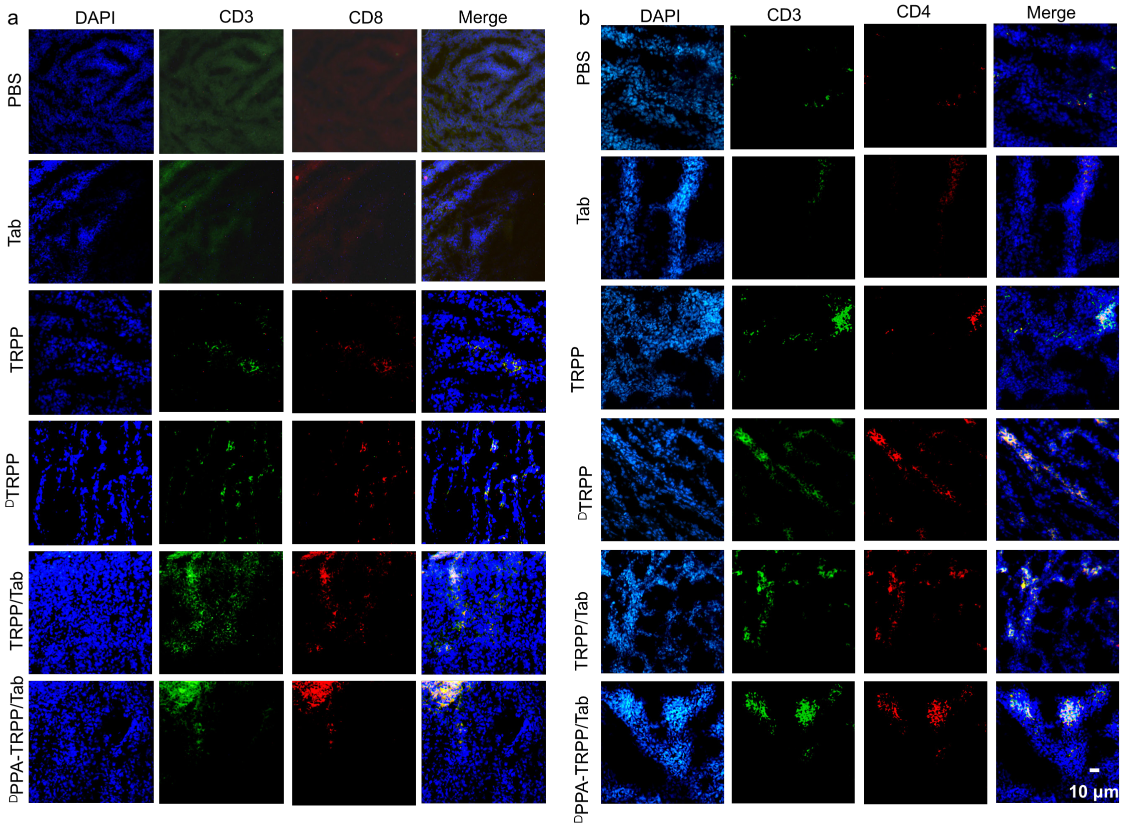

Figure S23. *In vivo* T cell infiltration for 4T1 tumor bearing mice at day 6 post treatment. (a) $CD8^+$ and (b) $CD4^+$ T cell infiltration *via* immunofluorescence staining.

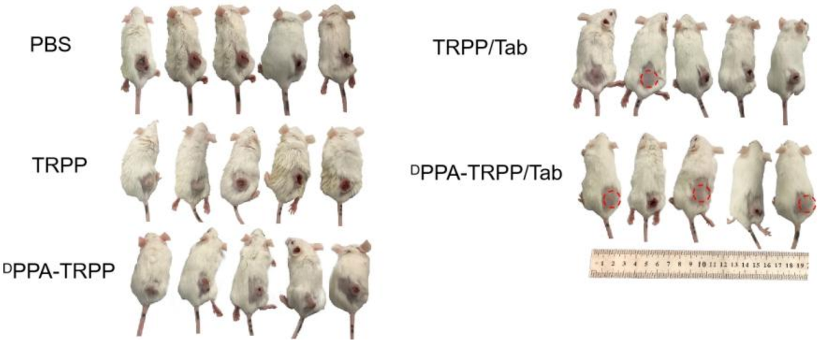

Figure S24. Tumor volume images for 4T1 tumor-bearing mice at day-27 post inoculation when the treatment started from day 6 after inoculation. Mice with red circle presented that tumor vanished after treatment.

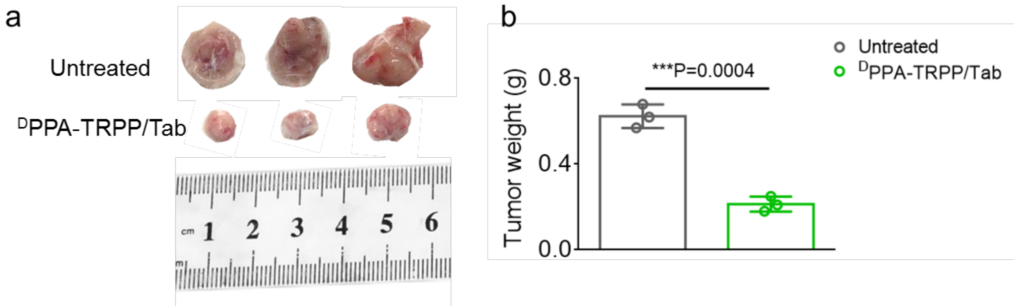

Figure S25. Tumor images (a) and tumor weight (b) for rechallenged mice at day 72.

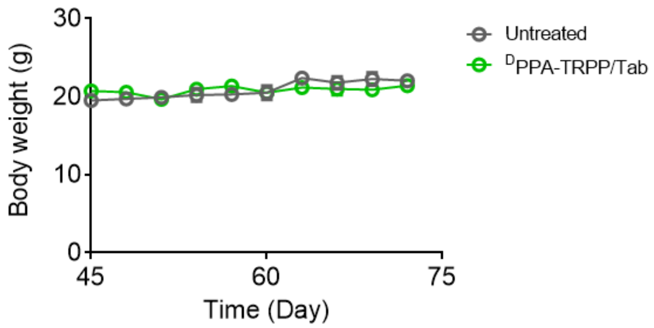

Figure S26. Body weight changes for rechallenged mice.

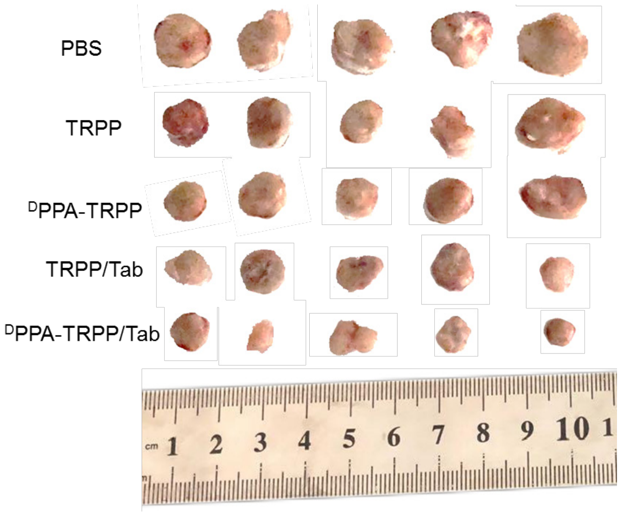

Figure S27. Tumor volume images for 4T1 tumor-bearing mice at the therapeutic ending point when the treatment started from day 9 after inoculation.

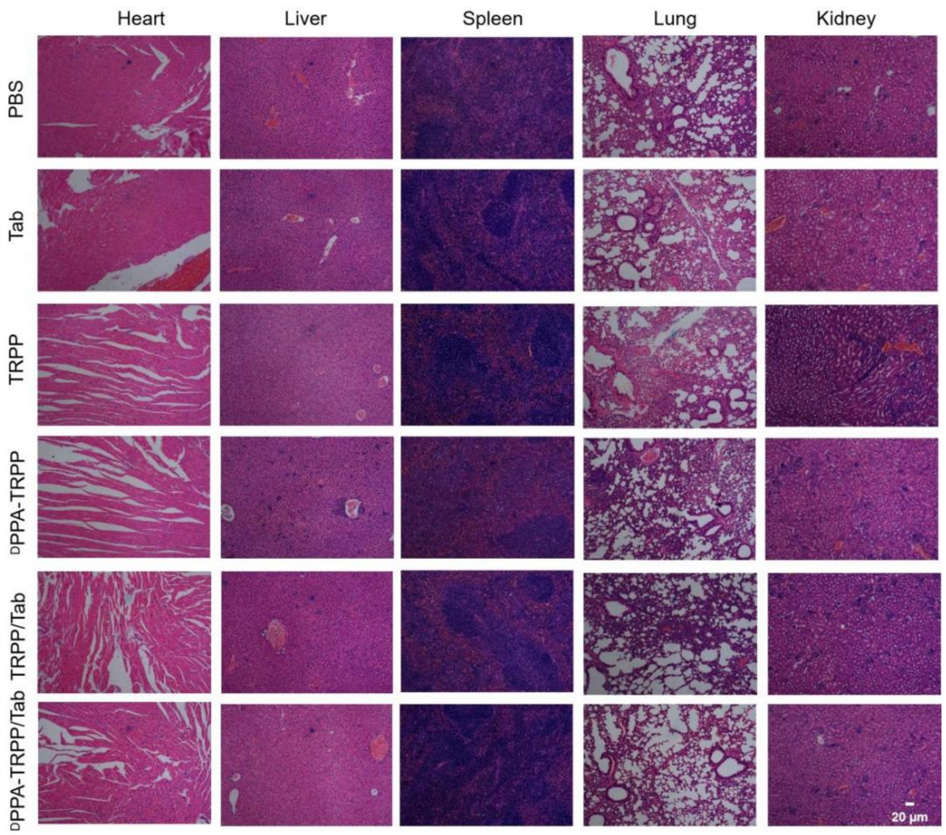

Figure S28. H&E results for the normal organs after treatment.

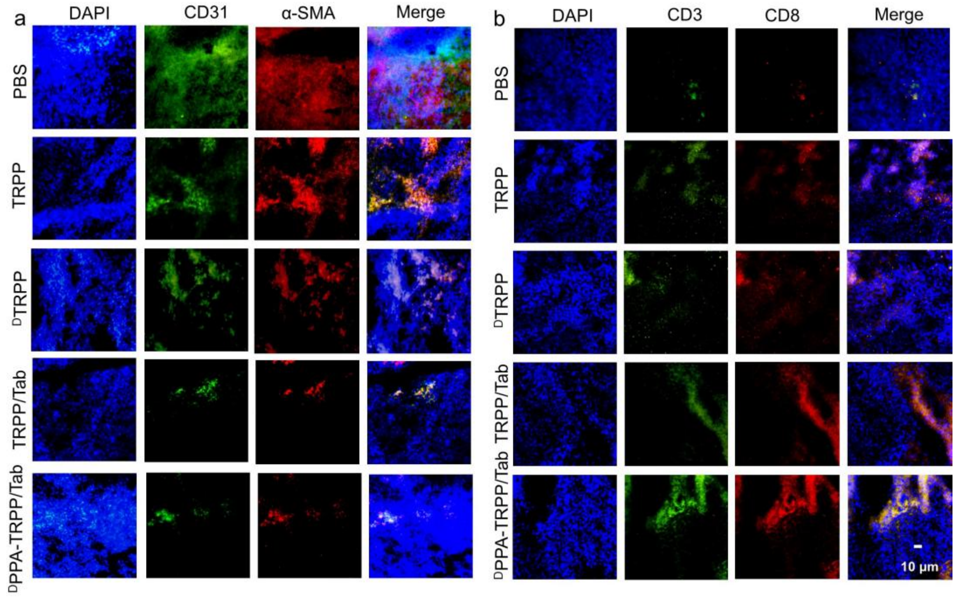

Figure S29. Immunofluorescence images for $CD8^+$ T cell infiltration and α-SMA expression at the therapeutic ending point for mice at day 9 post-treatment. TME modulation with (a) α-SMA down regulation, and (b) $CD8^+$ T cell infiltration.
